# A comparison of per sample global scaling and per gene normalization methods for differential expression analysis of RNA-seq data

**DOI:** 10.1371/journal.pone.0176185

**Published:** 2017-05-01

**Authors:** Xiaohong Li, Guy N. Brock, Eric C. Rouchka, Nigel G. F. Cooper, Dongfeng Wu, Timothy E. O’Toole, Ryan S. Gill, Abdallah M. Eteleeb, Liz O’Brien, Shesh N. Rai

**Affiliations:** 1 Department of Bioinformatics and Biostatistics, University of Louisville, Louisville, KY, United States of America; 2 Department of Anatomical Sciences and Neurobiology, University of Louisville, Louisville, KY, United States of America; 3 Department of Biomedical Informatics, Ohio State University, Columbus, OH, United States of America; 4 Department of Computer Engineering Computer Science, University of Louisville, Louisville, KY, United States of America; 5 Department of Cardiology, University of Louisville, Louisville, KY, United States of America; 6 Department of Mathematics, University of Louisville, Louisville, KY, United States of America; 7 Department of Internal Medicine, Oncology Division, Washington University, St. Louis, MO, United States of America; 8 Department of Epidemiology, University of Louisville, Louisville, KY, United States of America; New Jersey Institute of Technology, UNITED STATES

## Abstract

Normalization is an essential step with considerable impact on high-throughput RNA sequencing (RNA-seq) data analysis. Although there are numerous methods for read count normalization, it remains a challenge to choose an optimal method due to multiple factors contributing to read count variability that affects the overall sensitivity and specificity. In order to properly determine the most appropriate normalization methods, it is critical to compare the performance and shortcomings of a representative set of normalization routines based on different dataset characteristics. Therefore, we set out to evaluate the performance of the commonly used methods (DESeq, TMM-edgeR, FPKM-CuffDiff, TC, Med UQ and FQ) and two new methods we propose: Med-pgQ2 and UQ-pgQ2 (per-gene normalization after per-sample median or upper-quartile global scaling). Our per-gene normalization approach allows for comparisons between conditions based on similar count levels. Using the benchmark Microarray Quality Control Project (MAQC) and simulated datasets, we performed differential gene expression analysis to evaluate these methods. When evaluating MAQC2 with two replicates, we observed that Med-pgQ2 and UQ-pgQ2 achieved a slightly higher area under the Receiver Operating Characteristic Curve (AUC), a specificity rate > 85%, the detection power > 92% and an actual false discovery rate (FDR) under 0.06 given the nominal FDR (≤0.05). Although the top commonly used methods (DESeq and TMM-edgeR) yield a higher power (>93%) for MAQC2 data, they trade off with a reduced specificity (<70%) and a slightly higher actual FDR than our proposed methods. In addition, the results from an analysis based on the qualitative characteristics of sample distribution for MAQC2 and human breast cancer datasets show that only our gene-wise normalization methods corrected data skewed towards lower read counts. However, when we evaluated MAQC3 with less variation in five replicates, all methods performed similarly. Thus, our proposed Med-pgQ2 and UQ-pgQ2 methods perform slightly better for differential gene analysis of RNA-seq data skewed towards lowly expressed read counts with high variation by improving specificity while maintaining a good detection power with a control of the nominal FDR level.

## Introduction

High-throughput RNA sequencing (RNA-seq) has become the preferred choice for gene expression studies due to technological advances allowing for increased transcriptome coverage and reduced cost. These improvements have enabled studies with a large range of applications including identification of alternative splicing isoforms [1–3], *de novo* transcript assembly to identify novel genes and isoforms [4–6], detection of single-nucleotide polymorphisms (SNPs) [7,8] and novel single nucleotide variants (SNVs) [9], and characterization of mRNA editing [10]. In addition, RNA-seq enables the detection of rare transcripts while allowing for high coverage of the genome, which cannot be identified as well by microarray technologies [11]. However, the most common and popular application of RNA-seq experiments is the identification of differentially expressed genes (DEGs) between two or more conditions. These DEGs may serve as biomarkers for clinical diagnosis, with possible implications for prevention, prognosis and treatment [12,13].

Currently, several sequencing platforms exist, which require similar sample pre-processing and subsequent analytical steps, as summarized by Zhang *et al*. [14]. Briefly, this RNA-seq workflow consists of three major steps: 1) RNA-seq library construction; 2) sequencing and mapping; and 3) normalization and statistical modeling to identify the DEGs or transcript isoforms. Following the second step, raw mapped reads generated by an aligner such as TopHat2 [15] are further normalized by a variety of methods, which generally include within-sample and between-sample normalization. Normalization is a crucial step in gene expression studies for both microarray and RNA-seq data [16–19].

In RNA-seq, the expression level of each mRNA transcript is measured by the total number of mapped fragmented transcripts, which is expected to directly correlate with its abundance level. The expected expression level of each transcript is limited by the sequencing depth or total number of reads, which is pre-determined by the experimental design and budget before sequencing. Since the expression level of the transcripts within the sample is dependent upon the other transcripts present [20], given a fixed total read count, higher expressed transcripts will have a greater proportion of total reads [19,21]. Furthermore, longer transcripts have more reads mapping to them compared with shorter transcripts of a similar expression level [22]. Therefore, a number of normalization methods for RNA-seq data have been proposed to correct for library size bias as well as length and GC-content bias. These methods include per-sample Total Counts (TC) implemented in *EDASeq* [23,24], per-sample Upper Quartile (UQ) implemented in *edgeR*, *Cufflink-Cuffdiff2 and EDASeq* [18,24–26], per-sample Median (Med) implemented in *EDASeq* [23,24], DESeq normalization (median-of-ratios) implemented in *DESeq and DESeq2* [27,28], Trimmed Mean of M values (TMM) implemented in *edgeR* [19], Full Quantile (FQ) implemented in *Aroma*.*light* [29,30], Reads Per Kilobase per Million mapped reads (RPKM) [21] and Fragments Per Kilobase per Million mapped fragments (FPKM) implemented in Cufflinks-CuffDiff and *Cufflinks-CuffDiff2* [26,31,32], normalization by control genes [18,33] and normalization by GC-content [24]. To correct for library size, most of these methods, including TC, UQ, Med, DESeq and TMM, use a common scaling factor per sample to normalize genes. Among these, UQ, Med, FQ and control gene normalization are techniques previously employed in microarray analysis.

Given the variety of read count normalization methods for RNA-seq analysis, it can be challenging for scientists to determine which method is optimal with regards to sensitivity and specificity due to a variety of factors such as read depth, biological variation and the number of biological replicates in the RNA-seq data. Previous studies comparing these methods for DEG analysis suggested the use of *DESeq* and TMM-*edgeR* packages based on the false positive rate and detection power [18,20,23,34–36]. However, while *DESeq* and TMM-*edgeR* were reported to have overall better performance, these studies also report the false discovery rate (FDR) was higher than the nominal FDR, leading to an inflated type I error rate. Therefore, in this study, we explore new normalization methods and find a slight improvement over the existing methods with the dual goals of maintaining a nominal FDR level and a good specificity rate.

RNA-seq data are obtained from complex experiments with a variety of technical variations across different conditions and adjustments made for read depth and other variation [33]. For example, the mean read counts of genes can range from less than one reads for lowly abundant genes to thousands or millions of reads for highly abundant genes. In order to correct for the variation of each gene across samples or conditions, we propose a two-step normalization procedure: correcting the read depth through quantile normalization per sample followed by per gene and per 100 reads normalization across samples. This idea is adapted from the normalization of one-color cDNA microarray and RPKM and FPKM in RNA-seq [16,17,21,31]. The reads of each gene per sample are scaled by Med or UQ normalization. Then, the Med or UQ-normalized reads of each gene per sample are further scaled by the median per 100 reads across conditions. Thus, the reads in each gene are similarly scaled, allowing for an accurate comparison of gene expression across conditions.

In this study, we evaluated our methods (Med-pgQ2 and UQ-pgQ2) along with the public available methods. We used the exact test with a negative binomial distribution from *edgeR* to identify DEGs for the normalization methods including TC, Med, UQ, FQ and our two proposed methods. We used *DESeq2* for DESeq normalization and *Cufflinks-Cuffdiff2* for FPKM normalization to test DEGs. The benchmark Microarray Quality Control Project (MAQC2 and MAQC3) datasets, simulated data and real human breast cancer RNA-seq data with a variety of properties were used to compare these methods.

## Materials and methods

### Normalization methods

#### Within-sample and between-sample normalization methods

Within-sample normalization enables the correction of expression level in each gene associated with other genes in the same sample. Since a long gene or transcript has more reads mapping to it compared to a short gene or transcript with a similar expression, length normalization is taken into consideration in some normalization methods. Currently, the most widely used methods, including both within-sample and between-sample normalization, are RPKM [21] and FPKM [31]. FPKM is used to count the reads of a fragment for paired-end RNA-seq data, which produces two mapped reads. However, the correction for the difference in gene length for analysis of DEGs could introduce a bias in per-gene variance especially for low abundance genes [22,23].

#### Within-sample normalization methods

Since the prominent variation of read counts for a gene between samples is due to differences in library size or sequencing depth, within-sample normalization of raw reads is critical for the comparison of these gene expression measures across experimental conditions. The simplest normalization method is TC normalization, which adjusts the raw reads of each transcript by the total library size per sample. However, the comparison of RNA-seq normalization methods shows that Med, UQ, TMM from *edgeR*, DESeq and FQ normalization methods are much better than TC [23]. One reason is that a small number of highly expressed genes can consume a significant amount of the total sequence [18]. To account for this feature, scaling factors are estimated from the data and are used to achieve within-sample normalization [18,23].

#### Med-pgQ2 and UQ-pgQ2 normalization methods that we propose

Since the variation among genes within a sample and the variation per-gene across samples due to the systematical bias need to be corrected in order to accurately identify DEGs between conditions, we propose two-step per-gene normalization methods called Med-pgQ2 and UQ-pgQ2.

In the following, we define statistical notations for characterizing different normalization techniques. For simplicity, we only consider the gene *g* (*g* = 1,…,*G*) in sample *j* (*j* = 1,…,*n*) where *G* is the total number of genes and *n* is the total number of samples. Let *Y*_*gj*_ be the number of observed reads mapped to a gene *g* for sample *j*, *N*_*j*_ be the total number of mapped reads for all genes in sample *j*, *N* be the total number of mapped reads across all samples, $\bar{N}$ be the mean of the reads across all samples, *u*_*gj*_ be the true and unknown expression level and *L*_*g*_ be the length of the specific gene *g*.

The above *N*_*j*_, *N* and $\bar{N}$ can be expressed as:

$$N_{j}=\sum_{g=1}^{G}Y_{gj},\;N=\sum_{j=1}^{n}N_{j},\;\mathrm{and}\;\bar{N}=\frac{\left(\sum_{j=1}^{n}N_{j}\right)}{n}.$$

In this study, we examine eight existing and two proposed normalization methods with detailed statistical notations as described in S1 Appendix.

Like microarray data analysis, the raw read counts of RNA-seq data are first preprocessed to remove all zero read counts across conditions before the normalization procedure. Thus, in the case of a balanced sample size design, genes with total raw read counts across conditions less than the number of sample replicates are filtered out. For data with an unbalanced sample size design, the gene with an average number of raw reads across conditions less than one are filtered out. In addition, a value of 0.1 is added to the raw counts for those genes to avoid zero read counts that are used for the following normalization as well as the other normalization methods. Our proposed methods include two steps described as follows:

#### Step 1: Median and Quantile normalization

**a. Median (Med) [23].** Let $Y_{gj}^{Med}$ be the median-normalized reads of gene *g* in sample *j*. Median normalization is based on all constitutive gene reads with positive counts for all samples. For each sample *j*, the normalization factor $q_{j}^{50}$ is the median (50^th^ percentile or 2^nd^ quartile) of the mapped reads of the genes in each sample after filtering out the genes with zero read counts across samples [18]. The observed *Y*_*gj*_ is scaled by $q_{j}^{50}$ per average of median reads across all samples $\left({\bar{N}}_{med}\right)$. $Y_{gj}^{Med}$ can be expressed as:

$$Y_{gj}^{Med}=\frac{Y_{gj}}{q_{j}^{50}}\times {\bar{N}}_{med}=\frac{Y_{gj}}{q_{j}^{50}/{\bar{N}}_{med}}.$$ (1)

**b. Upper Quartile (UQ) [18].** If the majority of genes have very low read counts in a RNA-seq experiment, upper-quartile normalization is preferred to median normalization (50^th^ percentile) [18]. Let $Y_{gj}^{UQ}$ be upper-quartile-normalized reads of gene *g* in sample *j*. Upper-quartile normalization is based on all constitutive gene reads with positive counts for all samples. For each sample *j*, the normalization factor $q_{j}^{75}$ is the upper-quartile (75^th^ percentile) of the mapped reads of the genes in the sample after filtering out the genes with zero read counts across samples. The observed *Y*_*gj*_ is scaled by $q_{j}^{75}$ per average of upper-quartile reads across all samples $\left({\bar{N}}_{uq}\right)$. $Y_{gj}^{UQ}$ can be expressed as:

$$Y_{gj}^{UQ}=\frac{Y_{gj}}{q_{j}^{75}}\times {\bar{N}}_{uq}=\frac{Y_{gj}}{q_{j}^{75}/{\bar{N}}_{uq}}.$$ (2)

A study in evaluation of statistical methods for normalization in RNA-seq experiments [18] demonstrated that upper-quartile normalization reduced bias in the estimation of DEGs relative to qRT-PCR without noticeably increasing the level of variability as compared to TC normalization.

#### Step 2: Per-gene normalization after per-sample global scaling (Med-pgQ2 and UQ-pgQ2) as follows

**a. Med-pgQ2:** let $Y_{gj}^{Med}$ be the expression value for gene *g* in sample *j* scaled by the median (Med) in Eq (1); let ${Q2}_{g}^{Med}$ be the median of gene *g* across samples after median normalization per sample. Thus, the new normalized counts $Y_{gj}^{Med-pgQ2}$ per gene and per 100 reads can be expressed as:

$$Y_{gj}^{Med-pgQ2}=\frac{Y_{gj}^{Med}}{{Q2}_{g}^{Med}}\times 100.$$ (3)

**b. UQ-pgQ2:** let $Y_{gj}^{UQ}$ be the expression value for gene *g* in sample *j* and normalized by UQ (75%) in Eq (2); let ${Q2}_{g}^{UQ}$ be the median of gene *g* across samples after UQ normalization. Thus, the new normalized counts $Y_{gj}^{UQ-pgQ2}$ per gene and per 100 reads can be expressed as:

$$Y_{gj}^{UQ-pgQ2}=\frac{Y_{gj}^{UQ}}{{Q2}_{g}^{UQ}}\times 100.$$ (4)

The multiplication of 100 reads is used for per-gene normalization, similarly approaching as RPKM and FPKM normalizations in which the normalized reads are obtained via multiplication of one million of reads after being scaled by the length of a transcript per kilobase and the total read counts per-sample.

### Statistical model and the exact test

A study by Robinson *et al*. [37] demonstrated that the exact test is the best method when the sample size is small, and results in achieving the nominal FDR compared to other methods such as the Wald test, the Likelihood Ratio test (LRT) and the asymptotic normal score test. In order to compare these normalization methods, we chose a negative binomial distribution to model and the exact test to identify DEGs for the majority of the methods using *edgeR*. The detailed descriptions are available in S1 Appendix: Statistical model and the exact test.

#### The negative binomial (NB) distribution

Briefly, *Y* ∼ *NB*(*u*,ф) is a random variable to model the observed read counts in RNA-seq data, where *Y* has mean *u* and dispersion ф. Its probability mass function (pmf), the expected value and the variance of *Y* are correspondingly:

$$f_{Y}(y|u,ф)=P\left(Y=y\right)=\left(\begin{array}{c} y+{ф}^{-1}-1\\ y \end{array}\right){\left(\frac{1}{uф+1}\right)}^{{ф}^{-1}}{\left(1-\frac{1}{uф+1}\right)}^{y},$$

$$E(Y)=u\;\mathrm{and}\;Var(Y)=u+u^{2}ф.$$ (5)

The above NB model utilizes the conventional parameterization called “NB2” [38]. The dispersion parameter ф in Eq (5) measures the extra variance of *Y* that a Poisson *(u)* distribution fails to describe. As ф goes to zero (ф*→0)*, the variance of *Y* converges to *u* in probability and the distribution of *f(y)* in (5) converges to the Poisson *(u)* distribution which was shown by Cameron and Trivedi [39].

### Datasets

#### 1. MAQC2 and MAQC3 datasets

MAQC2 contains two RNA-seq datasets from the Microarray Quality Control Project (MAQC) [40] with two types of biological samples: human brain reference RNA (hbr) and universal human reference RNA (uhr). The first dataset consisted of read length of 36bp and was downloaded from the NCBI sequence read archive (SRA) with ID SRX016359 (hbr) and SRX016367 (uhr) [18]. The second dataset (GEO series GSE24284) consisted of the 50bp hbr (sample ID: GSM597210) and uhr (sample ID: GSM597211) RNA samples [41].

GSE49712_HTSeq.txt.gz for MAQC3 raw read counts with five technical replicates in two biological conditions (UHR and HBR) was downloaded from GEO (GSE49712) [20]. Four replicate libraries for two conditions were prepared by one person and the remaining library was prepared by Illumina. A single HiSeq2000 instrument was used for sequencing all the samples.

#### 2. TaqMan qRT-PCR data

PCR validation of the uhr sample from GSM12638 to GSM129641 and the hbr sample from GSM129642 to GSM129645 were downloaded from GEO (series GSE5350). These MAQC data (uhr and hbr) contain a total of 1044 genes assayed and validated using TaqMan qRT-PCR with 4 technical replicates [18,41]. Thirty-seven of the 1,044 genes were marked with a Flag Detection “A” in all samples and were considered as true negative (TN) genes. These additional genes were not filtered out as in recent studies of the MAQC validation datasets [42] and 1028 of the 1044 genes have either a unique Ensembl gene Identifier (ID) or Entrez gene ID used for further analysis of the true positive and true negative genes following Bullard *et al*.’s study [18]. Briefly, a POLR2A-normalized cycle number for each gene and each condition is called ΔCt. The value *x*_*gik*_ of each gene *g* in replicate *i* and condition *k* is obtained via *log*_2_(ΔCt)/*log*_2_(*e*). The log_2_ fold change is defined as the mean difference of each gene between the hbr and uhr conditions (${\bar{x}}_{g,hbr}-{\bar{x}}_{g,uhr}$), where the uhr is typically served as a reference. The genes with |log_2_ *FC*|≥2 were considered DEGs and the genes with |log_2_ *FC*|<0.2 were considered as non-DEGs. Among the 1028 genes, 398 genes with 390 unique gene names fall into the true positive (TP) genes and 178 genes with 151 unique gene names fall into the true negative (TN) genes. The remaining set of genes lie in a region set to be indeterminate as far as DEG is concerned.

#### 3. Two human breast cancer RNA-seq datasets

Dataset one is used for simulation containing twenty-four normal tissues and 25 early breast neoplasia (EN) on formalin-fixed paraffin-embedded tissue were sequenced at 3’-end enriched RNA-seq libraries [43].The mapped raw counts of 49 samples with an average of 7 million reads per sample were downloaded from NCBI GEO (series GSE47462). The dataset two contains 42 human estrogen receptor positive (ER+) and HER2 negative breast cancer primary tumors and 30 uninvolved breast tissues samples adjacent to ER+ primary tumors. The RNA-seq raw data files with a sequence read archive (SRA) were downloaded from NCBI GEO (GSE58135).

#### 4. Simulated data

Simulated data was based on the human breast cancer RNA-seq dataset one with two conditions: 24 normal tissues and 25 early neoplasia tissues. The simulation model is similar to the one described in Dillies’s study [23]. Let *G* be the total number of genes (*G* = 15,000), *n* = 20 be the total number of samples in two conditions (*k = A*, *B*), let *y*_*igk*_ be the count for gene *g* in sample *i* and condition *k* with a Poisson distribution: *y*_*igk*_ ∽ *Pois*(*λ*_*gk*_). The parameter *λ*_*gk*_ is estimated from the mean reads per gene across samples from this human breast cancer RNA-seq dataset. Under this model, the null hypothesis *H*_0_ (*λ*_*gA*_ = *λ*_*gB*_) means the expression values of gene *g* between conditions A and B are not significantly different, and the alternative hypothesis *H*_1_ (*λ*_*gA*_ ≠ *λ*_*gB*_) means the gene expression values are significantly different between the two conditions. Let *p*_0_ and *p*_1_ be the proportion of genes generated under *H*_0_ and *H*_1_ among the *G* genes, respectively. The data is simulated with 15,000 genes and *p*_1_ is 10% corresponding to 1,500 genes. Under *H*_0_, the parameter *λ*_*gA*_ in the gene *g* of condition *A* and the parameter *λ*_*gB*_ in the gene *g* of condition *B* were estimated from the breast cancer raw counts corresponding to the mean raw counts of each gene (*λ*_*gA*_ = *λ*_*gB*_); while under *H*_1_ the parameters *λ*_*gA*_ and *λ*_*gB*_ in the gene *g* and condition *A* and *B* were equal to (1 + *α*)*λ*_*gA*_ for 750 downregulated genes and (1 + *α*)*λ*_*gB*_ for 750 upregulated genes, respectively, where *α* is defined as 0.5 and 1. To assess the impact of non-equivalent library sizes, we multiplied *y*_*igk*_ by a size factor *S*_*i*_ per sample of the condition, which is equal to |*ε*_*i*_|, where *ε*_*i*_ ∼ *N*(1,1). The number of simulation was chosen as 13 due to the small variation of the AUC values from all the normalization methods per simulation.

### Sequence mapping and extraction of gene counts

The MAQC2 RNA-seq libraries with two technical replicates of each sample (uhr and hbr) and the human ER+ breast cancer dataset two were mapped to the human hg19 reference genome using *tophat2* (v2.0.13) with Bowtie version (2.2.3.0) and the parameter: ‘no-coverage-search’ [26,31]. For the FPKM normalization method, the aligned RNA-seq reads were assembled according to the Homo_sapiens.GRCh37.74.gtf annotation file and normalized by FPKM using *Cufflinks-Cuffnorm* (v2.2.1). For the other normalization methods, the aligned RNA-seq reads were sorted by *samtools* (v0.1.19) and the read count matrix for each replicate of the condition was generated using HTSeq-scripts-count (version 2.7) and provided in S1 Datasets. In addition, for the human ER+ breast cancer dataset, the read counts from two human ER+ breast cancer samples and one control sample failed to be extracted using HTSeq-script-count. Therefore, only 40 ER+ breast cancer and 29 control samples were used for this study.

### Software packages for detecting DEGs in normalization methods

The normalization methods and the software packages for detecting DEGs between conditions using MAQC datasets and the human ER+ breast cancer dataset are summarized in Table 1. Here, we give a brief description of the software packages used for the normalization and statistical tests in the present work. *edgeR* (v3.8.6) [25] was used to perform TMM normalization. It uses the empirical Bayes estimation and the exact test with a negative binomial distribution. For this study, edgeR was used to detect DEGs for all the seven normalization methods including TC, Med, UQ, FQ, TMM, Med-pgQ2 and UQ-pgQ2. *DESeq2* [28], a successor to the *DESeq* method [27], shows higher sensitivity and precision compared to DESeq package due to new features using shrinkage estimators for dispersion and fold changes. DESeq2 also offers a scaling size factor procedure as DESeq to perform normalization which is based on a median of ratio method. *Cufflinks-Cuffnorm* (v2.2.1) with a default parameter setting was used to perform FPKM normalization. Cufflinks-Cuffdiff2 was used to perform DEGs analysis at both the transcript and gene level using a beta negative binomial model and the t-test for the fragment counts [26]. In this study, we used the gene level results for the comparison with the other normalization methods. With the aid of edgeR, we set the normalization methods to “none” and selected the exact test with a tag-wise dispersion for each gene to perform DEGs analysis for the normalization methods: TC, Med, UQ, FQ, Med-pgQ2 and UQ-pgQ2. The normalized MAQC2 data from Med-pgQ2, UQ-pgQ2, DESeq and TMM-edgeR and DEGs analysis from these methods are also provided in Supporting Information S2 Datasets–S5 Datasets. Moreover, these normalization methods are written in R (v3.1.3) with the source codes available in S1 File (.R).

**Table 1:** Summary of normalization methods and software packages on different datasets for DEGs analysis.

Normalization methods	Datasets	Statistical test	Software packages
**TC**	MAQC and simulated data	Exact test	edgeR(v3.8.6)
**Med**	MAQC and simulated data	Exact test	edgeR (v3.8.6)
**UQ**	MAQC and simulated data	Exact test	edgeR (v3.8.6)
**FQ**	MAQC and simulated data	Exact test	edgeR (v3.8.6)
**TMM**	MAQC and simulated data	Exact test	edgeR (v3.8.6)
**Med-pgQ2**	MAQC and simulated data	Exact test	edgeR (v3.8.6)
**UQ-pgQ2**	MAQC and simulated data	Exact test	edgeR (v3.8.6)
**DESeq**	MAQC and simulated data	Wald test	DESeq2 (v1.6.3)
**FPKM**	MAQC	t-test	Cufflinks-cuffdiff2 (v2.2.1)

### The AUC, standard error and z-statistic test for MAQC data

The area under the ROC curve (AUC) was calculated using Algorithm 2 by Fawcett (2006) [44]. The estimated standard error (se) and a two-sample one-sided z-test were computed for each AUC value in MAQC data using Hanley J.A. *et al*. method (1982) [45]. Briefly, let *A* be the area under ROC curve; $\hat{se}$ and *sd* be the estimated standard error and standard deviation, respectively; *na* and *nn* be the total number of true positive genes and false positive genes, respectively. Then, $\hat{se}=\sqrt{d1/(na\times nn)}$, where *d*1 = *A* × (1 − *A*) + (*na* − 1) × (*Q*1 − *A*^2^) + (*nn* − 1)(*Q*2 − *A*^2^), $Q1=\frac{A}{2-A},\;Q2=2\times \frac{A^{2}}{1+A}$. The *Z* statistic was computed as: $z=\frac{A_{1}-A_{2}}{\sqrt{{\hat{se}}_{1}^{2}+{\hat{se}}_{2}^{2}}}$ and *p*.*value* = 1 − *Prob*(*Z* < *z*). This p-value was used to compare the AUC values between two normalization methods.

### The 95% confidence interval estimation of AUC for the simulated data

The 95% CI (confidence interval) for the simulated data was computed based on the normal approximation, which is defined as $CI=\bar{A}\mp 1.96\times \frac{sd}{\sqrt{n}}$, where *n = 13* is the number of simulations, $\bar{A}$ and *sd* are the mean and standard deviation of AUC from 13 simulations, respectively.

## Results and discussion

In this study, seven different normalization methods were compared to our proposed methods (Med-pgQ2 and UQ-pgQ2) via the qualitative characteristics of data distributions, intra-condition variation, ROC curve and AUC value as well as PPV, the actual FDR, sensitivity and specificity given the nominal FDR (≤ 0.05).

### Qualitative characteristics of data distributions

**Fig 1:**
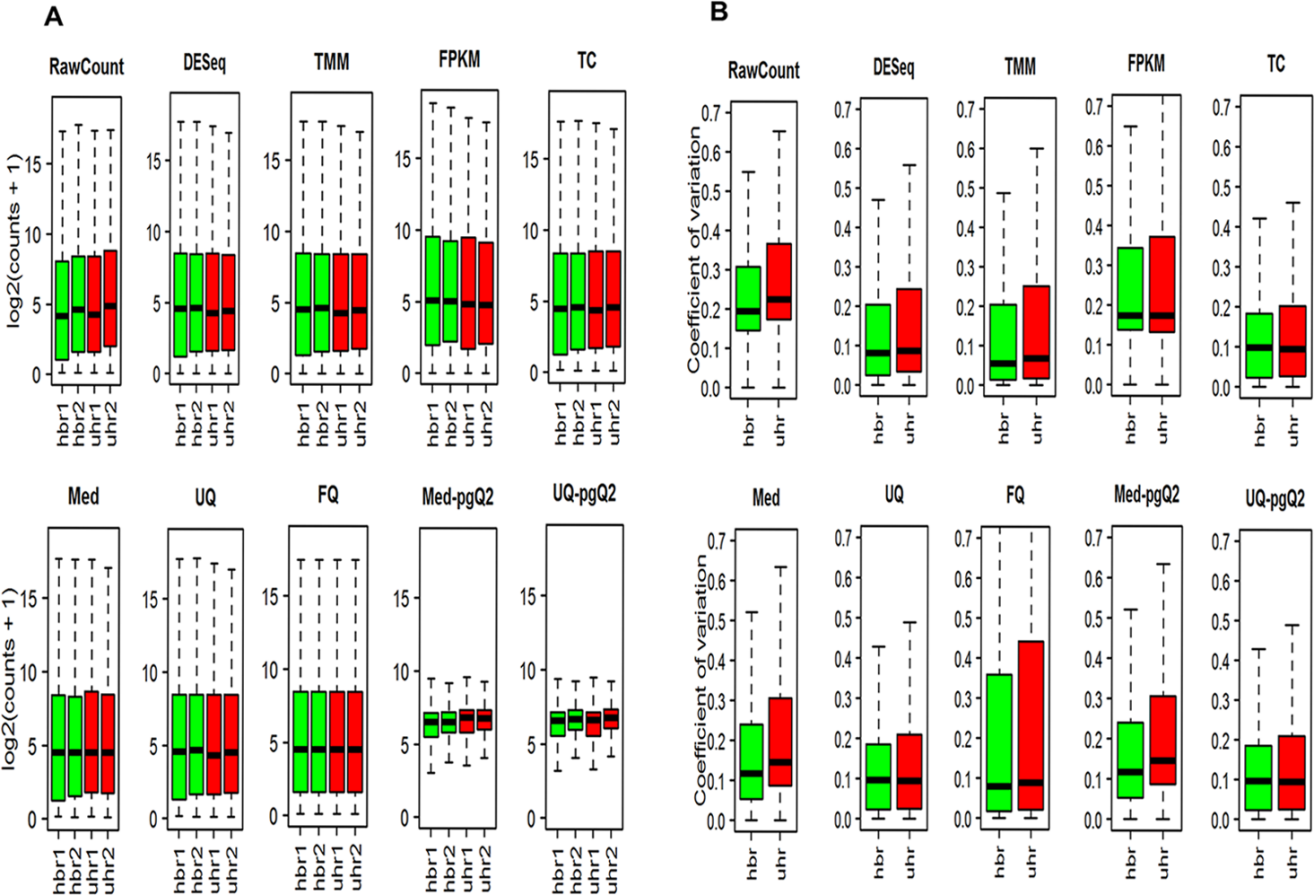
Comparison of nine normalization methods. (A) Illustrated are boxplots of log_2_ (counts+1) for MAQC data with two replicates in two conditions (uhr and hbr). The samples in hbr and uhr conditions are in green and red, respectively. Med-pgQ2 and UQ-pgQ2 are our proposed methods. (B) Illustrated are boxplots of the intra-condition coefficient of variation (uhr and hbr), respectively.

In DEGs analysis, one important assumption of null hypothesis about normalized RNA-seq data is that the majority of genes are not differentially expressed between conditions. Therefore, the overall distributions across genes are expected to be similar. Boxplots of non-normalized log_2_ expression of raw read counts in Fig 1A shows larger distributional difference between the replicate libraries for MAQC2 data and normalization methods are needed to make the sample distributions more similar. Although all the normalization methods stabilized the distributions across two replicates for MAQC2 data, only our methods further can shrink the gene expression values towards the median per sample (Fig 1A).

It is important to compare the intra-condition variation among different normalization methods to prevent over correction. Fig 1B from MAQC2 data illustrates that little difference of the intra-condition variation is observed between our methods and others (DESeq, TMM, TC, Med and UQ), which indicates that scaling does not change the coefficient of variation. Moreover, we observed that FQ and FPKM methods greatly increased the intra-condition variation compared to the un-normalized data and other normalization methods (Fig 1B). This observation was also reported by Dillies’ study in 2012.

We further analyzed the human ER+ breast cancer RNA-seq dataset with 40 ER+ breast cancer samples and 29 controls and MAQC3 with five technical replicates. For the human breast cancer datasets, similar patterns for most of the normalization methods from the boxplots (S1 and S2 Figs) are observed compared to MAQC2 in Fig 1. However, the intra-condition variation of the median across replicates for all the methods (S2 Fig) is close to 0.5, which is much higher than the value below 0.1 for all the methods obtained from the MAQC2 data (Fig 1B). This is expected because the breast cancer data contain biological replicates. We found that TC normalization failed in correcting the raw read counts for some of the replicates with a higher distributional difference within conditional replicates (S1 Fig). The failed TC normalization was also observed by Dillies’ study in 2012 using mouse miRNA-seq data. Furthermore, we also discovered that the inability of FQ normalization to minimize the intra-condition variation due to the small sample size from MAQC2 was diminished for the human ER+ breast cancer datasets with the sample size of 29 in control and 40 in ER+ breast cancer samples (S2 Fig).

For MAQC3 data, the boxplots (S3 Fig) show that sample distributions normalized by all methods are very similar, which is expected due to technical replicates with very small variation. These data with less variation after scaling normalization suggest that a further per gene normalization may not show a great advantage other than shrinking the data toward the median across samples.

### RMSD between qRT-PCR and RNA-seq *log*_2_ fold change computed by each method

**Fig 2:**
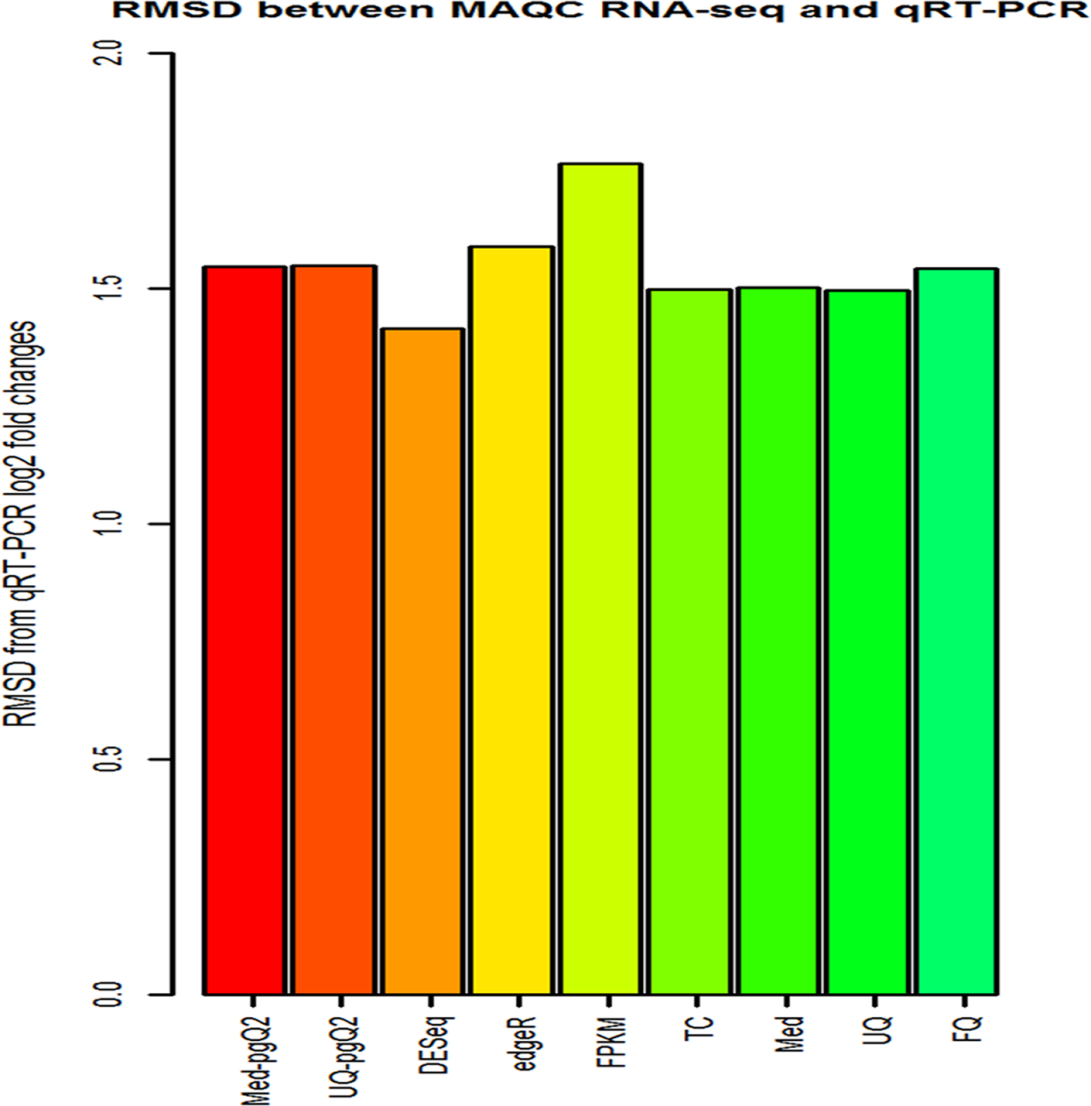
RMSD (root-mean-square deviation) between the log_2_ expression fold changes of MAQC2 and qRT-PCR. Illustrated is the RMSD between the log_2_ fold changes computed from DEGs based on different methods and the values computed from qRT-PCR. FPKM (yellow) has the least similarity while DESeq normalization (brown) has the highest one.

To evaluate the accuracy of normalization methods, we used the MAQC2 and qRT-PCR data to calculate RMSD (root-mean-square-deviation) correlation between the log_2_ fold changes generated from statistical tests for each normalization method (Table 1) and the log_2_ fold changes from qRT-PCR. Fig 2 illustrates that almost all the normalization methods have good concordance to match the qRT-PCR data with RMSD accuracy less than 1.6 except *Cufflinks-Cuffdiff2* with a slightly higher RMSD value (1.77).

### Analysis of differentially expressed genes evaluated by ROC curves and AUC values

**Fig 3:**
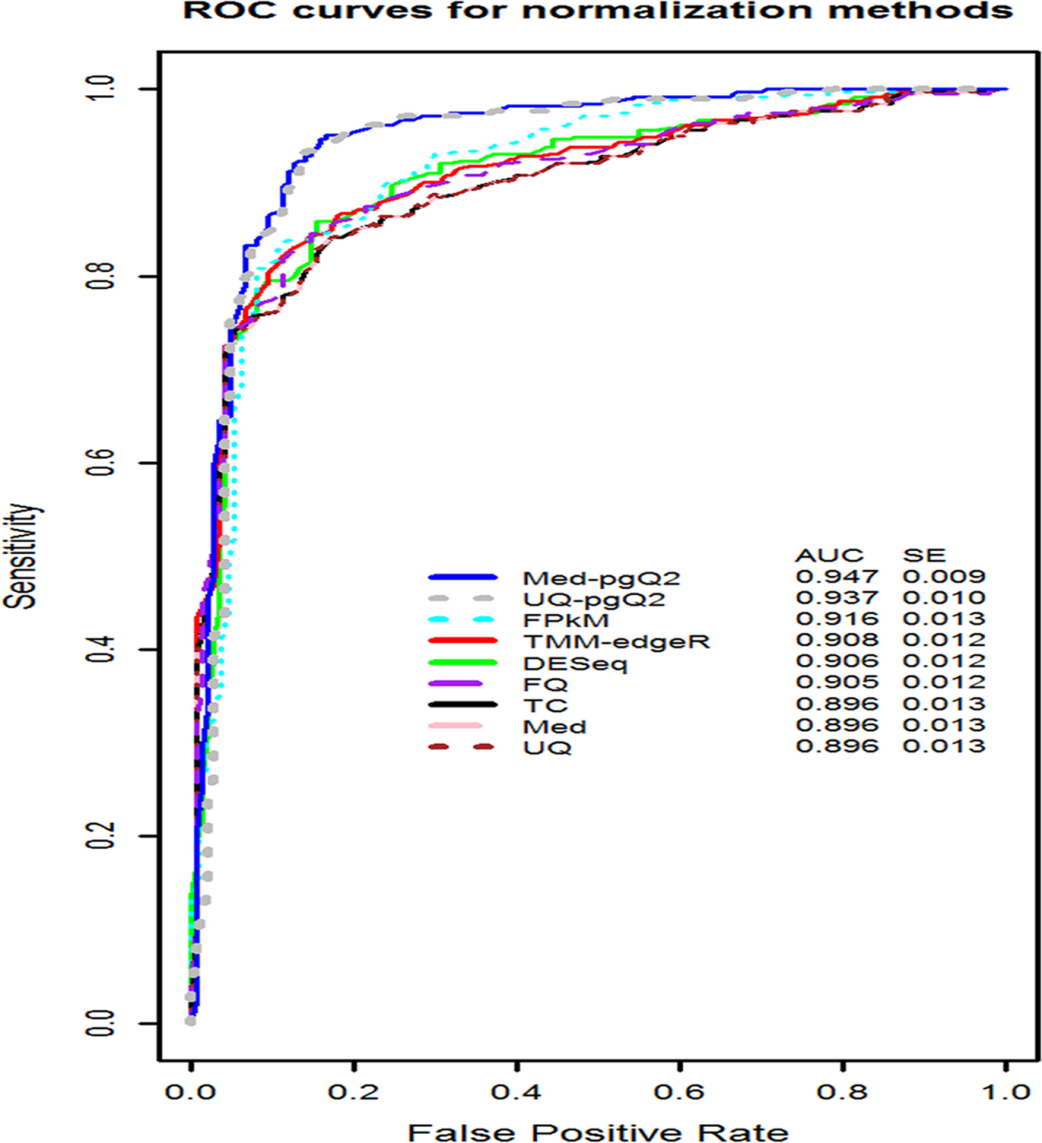
ROC curve and AUC values from MAQC2 data. The ROC curves and AUC values (inset) for evaluating the performance of the nine normalization methods were computed using MAQC2 with two conditions (uhr and hbr). Our proposed methods, Med-pgQ2 and UQ-pgQ2 (blue and grey, respectively) performed slightly better.

The ROC curve in Fig 3 is depicted by the relationship between the sensitivity and specificity rate based on MAQC2 data. The AUC value is calculated in the full range of false positive rate (0≤ FPR ≤1). Med-pgQ2 and UQ-pgQ2 achieve slightly higher AUC values compared to the others, which reflects the overall performance of detection of DEGs by achieving slightly higher sensitivity and specificity. With a false positive rate ≥ 0.10, the ROC cures reveal our methods perform slightly better. However, with a higher stringent false positive rate cutoff (< 0.10), the majority of the methods perform similarly. The quantile global normalization methods including TC, Med, UQ and FQ perform less favorable for this data. The standard error corresponding to the AUC value was also calculated using the equation from Hanley *et al*. in 1982 [45].

In addition, we further compared the AUC value from one of our methods (Med-pgQ2) to the others using a two-sample one-sided *z*-test. Table 2 lists the results of the p-values for each method. The results demonstrate statistically significant evidence that the AUC value in Med-pgQ2 is slightly larger than every other method except UQ-pgQ2.

**Table 2:** A one-sided of z-test on AUC values from Fig 3 comparing Med-pgQ2 to other methods.

	UQ-pgQ2	FPKM	TMM	DESeq	FQ	TC
**z-statistics**	0.7554	2.0082	2.0096	2.5826	2.6861	2.7517
p-value*	0.2250	0.0223	0.02224	0.0049	0.0036	0.0030

*p-values were computed using a one-sided of z-statistic test on the AUC values between Med-pgQ2 and one of the other methods listed in Table 2.

### Analysis of PPV, actual FDR, sensitivity, specificity, and the number of true positive and false positive genes

In order to identify the major difference among all the normalization methods for detection of DEGs in MAQC2 and MAQC3 data, we calculated the number of true positive (TP) genes and false positive (FP) genes given the nominal FDR ≤ 0.05. We also calculated the positive predictive value (PPV), the actual false discovery rate (FDR), sensitivity and specificity for both datasets (Table 3). The results from MAQC2 data suggest that Med-pgQ2 and UQ-pgQ2 can achieve better specificity rate above 85% than other methods. While TMM-edgeR has the highest sensitivity rate (96.7%), its specificity rate (35%) is low. The performance of DESeq normalization with the sensitivity and specificity rate at 93.1% and 60.9% correspondingly are between our methods and TMM. The two proposed methods also achieve the lower actual FDR (< 0.1) compared to others. However, the results from MAQC3 with small variation in Table 3 show that all the methods achieve very high sensitivity rate above 98%, but the specificity for all the methods is lower than 42% and the actual FDR is higher than 0.15. The two new methods for these data perform slightly better in term of sensitivity, specificity and the actual FDR.

**Table 3:** Analysis of DEGs for MAQC2 and MAQC3 given a nominal FDR ≤ 0.05.

Datasets	Methods	# of TP genes	# of FP genes	Actual FDR	PPV	SR	SPR
**MAQC2**	DESeq	363	59	.140	.860	.931	.609
	TMM	377	97	.204	.797	.967	.358
	FQ	377	100	.210	.790	.967	.338
	TC, Med & UQ	376	101	.212	.788	.964	.331
	Med-pgQ2	362	22	.057	.942	.928	.854
	UQ-pgQ2	364	21	.055	.945	.933	.861
**MAQC3**	DESeq	385	105	.214	.786	.990	.271
	TMM	385	98	.203	.797	.990	.319
	TC, Med & UQ	384	99	.204	.795	.987	.313
	Med-pgQ2 & UQ-pgQ2	387	83	.177	.823	.995	.424

The number of true positive (TP) and the false positive (FP) genes, the actual false discovery rate (FDR), the positive predictive value (PPV), the sensitivity rate (SR) and specificity rate (SPR).

We further analyzed the DEGs detected only by the top performers such as DESeq, TMM and our methods using different quartile cutoff of mean expression of raw read counts from all genes given the nominal FDR ≤ 0.05. The results for the actual FDR, sensitivity and specificity are listed in Table 4. With the quantile cutoff at 75% by keeping the bottom reads in the analysis, the DESeq normalization has slightly better values in term of the actual FDR and specificity rate than other methods. TMM is least favorable in this case. With the quantile cutoff at 50%, DESeq outperforms others. With the quantile cutoff at 25%, TMM shows better performance than others and DESeq is relatively conserved. However, since there are a fewer genes listed as true positive and true negative genes at the quantile cutoff at 25% in MAQC2 data, this conclusion is not arbitrary. However, Table 4 suggests that our proposed methods (Med-pgQ2 and UQ-pgQ2) at the 100% quantile can achieve a sensitivity and specificity rate higher than 92% and 85% with the actual FDR less than 0.06, respectively. This study based on the MAQC2 data suggests our methods can improve specificity rate and the actual FDR for highly expressed genes. Based on the overall performance, it clearly indicates our methods might be the better choice for this kind of data.

**Table 4:** The actual FDR, sensitivity and specificity rate from MAQC2 data given a nominal FDR ≤ 0.05.

Expression quantile cutoff	DESeq			TMM-edgeR			Med-pgQ2			UQ-pgQ2		
	Actual FDR	SR	SPR	Actual FDR	SR	SPR	Actual FDR	SR	SPR	Actual FDR	SR	SPR
100%(total)	0.140	0.931	0.609	0.205	0.967	0.358	0.057	0.928	0.854	0.055	0.933	0.861
75%	0.069	0.861	0.806	0.147	0.931	0.516	0.084	0.877	0.758	0.077	0.898	0.774
50%	0.091	0.476	0.926	0.184	0.738	0.740	0.304	0.762	0.482	0.292	0.810	0.482
25%	0.000	0.000	1.000	0.333	0.333	0.917	0.667	0.667	0.333	0.692	0.667	0.250

The sensitivity rate (SR) and specificity rate (SPR) for DEGs analysis by the top methods at the different-quartile cutoffs.

To address the question of how gene-wise normalization methods (Med-pgQ and UQ-pgQ2) improve specificity while maintaining good detection power for highly expressed genes, we further analyzed gene-wise dispersion estimated after UQ and UQ-pgQ2 normalization with the aid of *edgeR* (Supplemental S4 Fig). Subsequently, gene-wise variance was estimated on the basis of the mean and estimated dispersion assuming a negative binomial distribution. We examined the coefficient of variation (CV) in two sets of genes based on a cutoff value of the mean read count (<100 vs. ≥100) from the UQ method. Genes with mean read count < 100 after UQ normalization were considered lowly expressed while the other genes were considered highly expressed. Supplemental S5 Fig shows that the coefficient of variation for highly expressed genes after gene-wise normalization is increased via increasing the gene-wise dispersion and decreasing the per-gene mean read count compared to UQ normalization. This suggests that per gene normalization is more conservative for highly expressed genes, which at least partially explains our observation of improved specificity for these genes (Table 4). On the other hand, the coefficient of variation in lowly expressed genes after gene-wise normalization is slightly decreased compared to UQ normalization (S4 Fig, bottom). This suggests that per gene normalization is less conservative for lowly expressed genes explaining our observation that our gene-wise normalization methods slightly improve sensitivity in this case (Table 4).

### Evaluation of normalization methods for detecting DEGs using different fold changes

**Fig 4:**
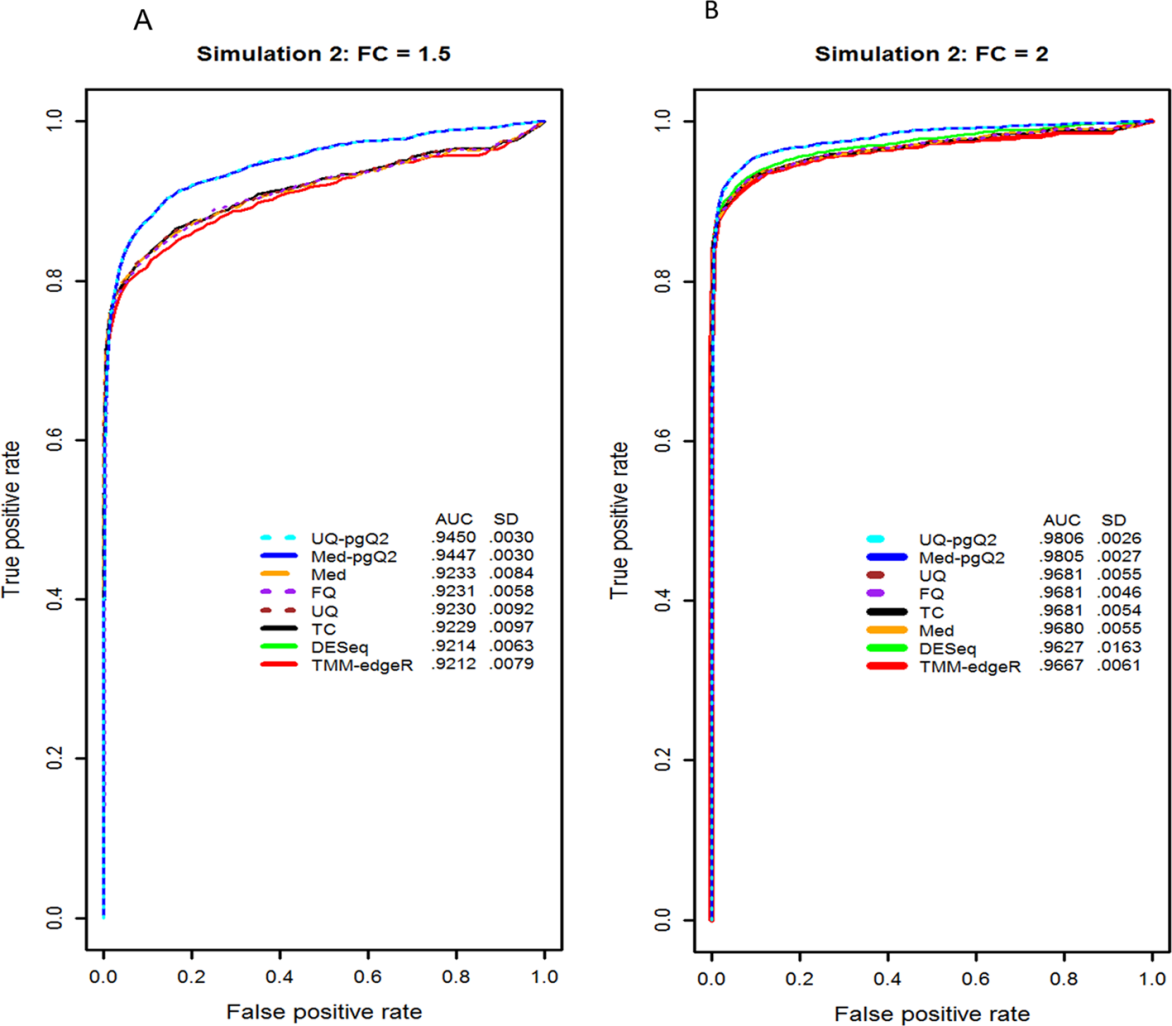
ROC curve and AUC values from the simulated data at a fold-change of 1.5 and 2. Illustrated are the ROC curves for detecting 1, 500 DEGs (750 up and 750 dow-regulated) using a fold change = 1.5 (A) and a fold change = 2 (B) with an unequal library size. Calculated AUC values are in the inset. The simulated data, containing a total of 15,000 genes in two conditions and 10 replicates per condition, was used for evaluating the performance of eight normalization methods. Our methods (UQ-pgQ2 and Med-pgQ2) are in cyan and blue, respectively.

The simulated data with 10 replicates and two conditions with different fold changes were used to compare our methods (Med-pgQ2 and UQ-pgQ2) based on the ROC curves. A total of 1,500 genes with a fold change (FC) of 1.5 and 2 are considered as true positive genes and the remaining genes (13,500) are considered as true negative genes. Fig 4A shows that the ROC curves for a FC of 1.5 in our methods have an average AUC value of 0.945 compared to others with the AUC value less than 0.924. Fig 4B shows that the ROC curves for a FC of 2 in our methods have the average AUC values greater than 0.980 compared to others with AUC values less than 0.969. However, the difference in the ROC curve and AUC values between our methods and others decreases as the fold change increases.

### Evaluation of normalization methods for detecting DEGs with biological replicates

We investigated the impact of biological replicates on the performance of normalization methods. We randomly sampled four and six replicates from each of 13 simulated datasets with 10 replicates used in Fig 4B, respectively. We sampled twice from one of 13 simulated data in Fig 4B yielding a total of 14 simulations. The mean AUC and standard deviation (SD) of each normalization method were calculated using 14 simulations instead of 13 simulations. The results from each simulation were consistent with a small standard deviation.

**Fig 5:**
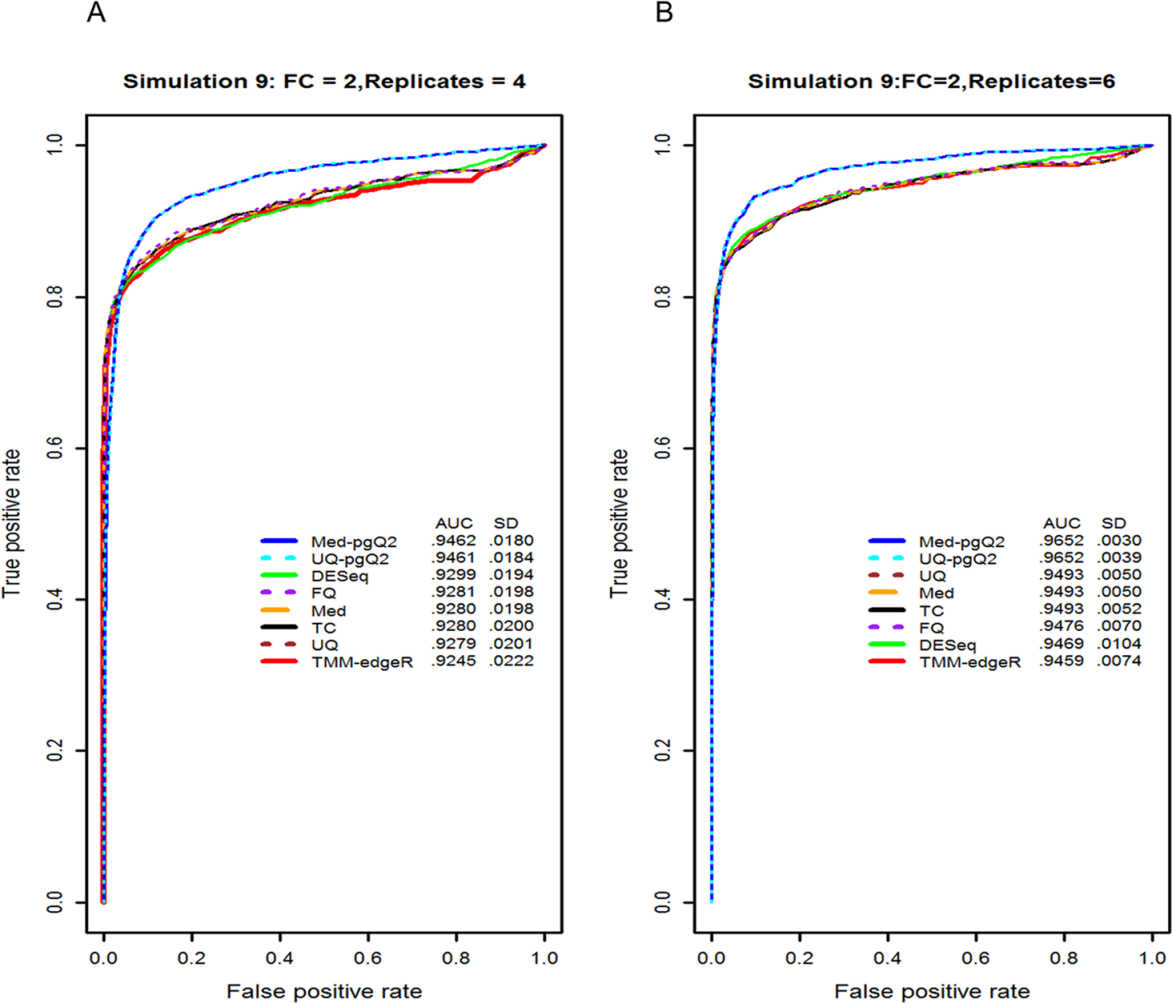
ROC curve and AUC values from the simulated data with 4 and 6 replicates in each condition. Illustrated are the ROC curves and AUC values (inset) in analyzing the impact of biological replicates on the performance of normalization methods. We used the simulated data with four biological replicates (A) and six biological replicates (B), which contain 1,500 DEGs with 2 FC difference between two conditions. Our methods (UQ-pgQ2 and Med-pgQ2) are in cyan and blue, respectively.

As expected, increasing the number of biological replicates yields a higher statistical power for detection of DEGs (Fig 5). Under the control of a very small false positive rate, the performance of all the methods (Med-pgQ2 and UQ-pgQ2) is similar. Fig 5 demonstrates that biological replicates are very important for RNA-seq data analysis in order to find true biological difference between conditions. Our normalization methods would be a good choice for achieving a slightly higher sensitivity rate at the false positive rate cutoff greater than 0.1. However, a closer examination for FPR cutoff less than 0.1 indicated that when the number of replicates is smaller (4 instead of 6), the other methods actually perform better than our proposed methods at a FPR cutoff less than 0.1 (Fig 5A). This suggests that per gene normalization does not perform well for all circumstances. Therefore, caution is needed when choosing an optimal normalization method by taking into consideration the number of different replicates and their variation.

### Evaluation of Med-pgQ2 and UQ-pgQ2 methods for detecting DEGs in different multiplication factors (50, 100, 200, 500, 1000 and 1 million)

Like RPKM and FPKM, we chose to use the small multiplication factor of 100 for our proposed per gene and per 100 normalization for this study. We also chose different multiplication factors such as 50, 200, 500, 1000 and 1 million to perform per gene normalization. We performed DEGs analysis using Med-pgQ2 and UQ-pgQ2 with these multiplication factors. The comparison results based on DEGs analysis are shown in S1 Table. We compared the impact of multiplication factors on PPV, the actual FDR, sensitivity and specificity. The values of PPV, the actual FDR, a sensitivity and specificity rate with multiplication factor ≥ 100 (S1 Table) are more than 94%, less than 0.06, more than 92% and more than 85%, respectively. Little difference among them is observed except with the multiplication factor of 50 having a slightly higher sensitivity rate with a trade-off of a slightly higher actual FDR and a lower specificity rate. These results suggest that the choice of multiplication factors with a value greater than or equal to 100 has no difference on DEG analysis results.

## Limitations

Our study has some limitations. First, the data normalized by med-pgQ2 and UQ-pgQ2 is restricted for DEGs analysis between groups and not for other purpose such as identifying highly or lowly expressed genes as well as comparing gene A to gene B expression levels within a sample due to the potential change of gene order in a sample after normalization. Second, a simulation data using a Poisson distribution based on real RNA-seq data with additional variation generated from a normal distribution was used for the DEG analysis. We do acknowledge that the lack of simulated data based on the NB distribution is a limitation to the study. However, inclusion of two real data sets (MAQC2 and MAQC3) offsets this limitation to an extent, and the combination of the simulated and real data provides fairly comprehensive and consistent answers. Finally, on one hand, the exact test was used to identify DEGs implemented by edgeR. Although it is recommended for DEG analysis of RNA-seq data in two groups with a small sample size, we think that evaluating the effect of normalization on more complicated study designs beyond two-group comparisons is a worthwhile and interesting endeavor, and we may consider this as potential future work. On the other hand, although a t-test is not commonly used for testing hypothesis in RNA-seq data, it is used for testing DEGs with small sample size in the cDNA Microarray data. Therefore, we need to mention here that a t-test is invariant to linear transformations and thus would be unaffected by the per-gene normalization outlined here.

## Summary and conclusion

Several studies have previously compared normalization methods (TC, Med, UQ, FQ, DESeq, TMM, FPKM and RPKM). TC, FPKM, RPKM and FQ are not suggested for use in DEG analysis due to multiple issues such as lowly expressed gene issue for TC, length correction bias for FPKM and RPKM, and potentially increasing the intra-condition variation by forcing all the samples to have identical distributions for FQ [18,20,22,23]. One study has reported that UQ normalization failed to remove excessive variation from some of the samples [33]. DESeq and TMM-edgeR are in turn the only choices due to better performance compared to other existing methods. Although DESeq appears relatively conservative compared to TMM-edgeR method [36,46], a high false-positive rate particularly for highly expressed genes for both methods has been observed by several studies [34,42].

In this study, we compared two new normalization methods for RNA-seq data analysis (Med-pgQ2 and UQ-pgQ2) to the seven existing methods (DESeq, TMM-edgeR, FPKM-CuffDiff, TC, Med, UQ and FQ) based on DEG analysis. The purpose of using per-gene normalization approach is to remove technical variations using different chips and allow for comparison between conditions based on similar count levels [47,48]. The results from this study demonstrate our proposed methods (Med-pgQ2 and UQ-pgQ2) can achieve a slightly higher value of AUC for both MAQC2 data and the simulated data at the false positive rate of 0.10, which reflects improving the overall performance with the detection power under the control of the low FDR compared to other normalization methods. More importantly, the results of DEG analysis from MAQC2 data with the different quantile cutoff values given a nominal FDR≤ 0.05, demonstrate our methods can decrease the false positive rate for highly expressed genes with high read counts giving the result of a specificity rate of greater than 85% without loss of a detection power (> 92%), while the other methods (i.e., DESeq and TMM-edgeR) have a specificity rate of less than 70%. Our methods may improve the sensitivity and detect more DEGs for lowly expressed genes with low read counts. However, given the improvement in the sensitivity for low read-count genes, there is a trade-off of a higher false positive rate in this case compared to DESeq and TMM-edgeR. Furthermore, the overall results from MAQC2 data also show the actual FDR from our methods is less than 0.06 while the actual FDR from DESeq, TMM-edgeR and others are larger than 0.10. This finding is consistent with the report by Kvam *et al*. in 2012. In their study they compared *DESeq*, *edgeR*, *baySeq* and *TSPM* (two-stage Poisson model) methods via a simulated data and reported the FDR in these methods are not controlled well and the actual FDR is larger than the observed FDR [34]. Moreover, we discovered DESeq and TMM have better overall performance than TC, Med, UQ and FQ, which is also consistent with previous studies. In addition, based on the quantile cutoff analysis of DEGs in MAQC2 data, we observed that DESeq is a good choice for moderately expressed genes at the quantile cutoff of 75%, but it is too conservative for lowly expressed genes at quantile cutoffs below 50%. However, TMM method seems to have better control of the false positive rate for the lowly expressed genes. In addition, the simulated study with four replicates shows that DESeq and TMM-edgeR methods perform better than our methods at the FPR cutoff less than 0.05. These new findings may give a better idea for the choice of different normalization methods.

There are several specific potential applications of our normalization methods worth mentioning. First, our methods may be useful for analyzing microRNA sequencing (miRNA-seq) data. Since miRNA expression is usually low compared to the mRNA with a ratio range 0.1~1.3% of total RNA in rat and mouse species, and 0.5~9.2% of total RNA in human samples, the data might be skewed to the low read counts. Therefore, per gene normalization may increase the sensitivity with a relative better specificity for detection of differentially expressed miRNAs [49,50]. However, a comparison study of the performance for analyzing miRNA-seq between our methods and TMM-*edgeR* is needed to make definitive conclusions. Second, our methods are more universally applicable than using control-gene normalization in removing technical variations since it is hard to identify control genes such as housekeeping genes that remain at the same expression level regardless of the experimental conditions [23]. Third, given the importance of downstream analysis on RNA-seq data with a choice of normalization methods, our methods might be useful, particularly in light of emerging single-cell RNA-seq data and meta-analysis of RNA-seq data which have highly variable properties.

Finally, the simulated data results show that increasing the number of the biological replicates results in higher ROC curves and AUC values corresponding to higher detection power and lower false positive rate. However, due to the cost of RNA-seq data, the sample size of biological replicates was not considered by some of the earlier researchers using NGS technologies. One study by Hansen et al. in 2011 summarized a large number of published RNA-seq studies with a table showing that most of them had only one or a few biological replicates [51]. The thousands of DEGs identified from these RNA-seq data lack confidence and require further validation. Although laboratory qRT-PCR and Western blotting methods can be used to validate these identified DEGs, it is very tedious and almost impossible to validate several thousand DEGs. Our per gene normalization methods may be useful for combining the single or a few replicates of RNA-seq data from different experiments with the same conditions to increase the power for DEGs analysis.

Like many normalization and pre-processing procedures, our methods involve several choices of constants which we evaluated empirically. Primarily, in the 2^nd^ step of our methods we chose to scale the median across samples to be per 100 reads instead of per kilobase or per million reads which was used by RPKM or FPKM. Our justification for this choice of a scaling constant in S1 Table shows little difference of PPV, the actual FDR, specificity and sensitivity for multiplication factors ≥ 100 from DEGs analysis, and we picked the smallest scaling factor possible for which this was true. Secondly, a small positive value (such as 0.1 of one read) is added in all gene counts to avoid undefined fold changes in DEGs due to zero counts possible in one condition. This ensures no missing value for DEGs analysis and reduces the variability at low count values [52]. To study the robustness of results in the analysis of MAQC2 data, we considered different additive values (0.05, 0.1, 0.15, 0.2, 0.3, 0.4 and 0.5). The results in S2 Table (Supplementary Information) suggest that the FDR and sensitivity rate monotonically increased and the specificity rate monotonically decreased as increase in the additive values. Small positive values such as 0.05, 0.10 and 0.20 are recommended as FDR is reasonably maintained (less than 10%) with sensitivity and specificity rates of at least 80%. Furthermore, it is worth mentioning that preprocessing RNA-seq data such as prefiltering zero read counts across groups or adding a small positive number to all gene read counts is an option in RNA-seq data analysis. For example, the procedure to prefilter zero read counts may not avoid filtering out the lowly expressed genes which may be of interest by some researchers. Therefore, the choice of preprocessing the data will vary according to the experimental study.

Taken together, with the regards to all the discussed limitations, we think our proposed gene-wise normalization methods (Med-pgQ2 and UQ-pgQ2) might be a good choice for the skewed RNA-seq data with high variation via improving the false positive rate and maintaining a good detection power for DEGs analysis of RNA-seq data compared to the other normalization methods.

## Supporting information

S1 Fig Data distribution from seven normalization methods using human ER+ breast cancer datasets. (PDF)

S2 Fig The intra-condition coefficient of variation using human ER+ breast cancer datasets. (PDF)

S3 Fig Data distribution from seven normalization methods using MAQC3 data. (PDF)

S4 Fig Mean vs. Dispersion after UQ and UQ-pgQ2 methods using MAQC2 data. Gene-wise dispersion was estimated after UQ and UQ-pgQ2 normalization with the aid of *edgeR*. The top graph displays mean versus gene dispersion for genes with a quantile cutoff value of mean read count after UQ normalization of ≤ 90%, while the bottom graph displays mean versus gene dispersion for genes with a quantile cutoff value of mean read count after UQ.pgQ2 normalization of ≤ 90%.
(PDF)

S5 Fig Coefficent of variation (CV) after UQ and UQ-pgQ2 methods using MAQC2 data. The calculated coefficient of variation (CV) after UQ normalization and per-gene (UQ.pgQ2) normalization, based on the estimated dispersion parameter from *edgeR* and assuming a negative binomial distribution. The top graph displays the CV for genes with mean read count after UQ normalization of ≥ 100, while the bottom graph displays the CV for genes with mean read count <100.
(PDF)

S1 Table The effect of the multiplication factors on DEGs analysis for Med-pgQ2 and UQ-pgQ2 given the nominal FDR≤0.05. The number of true positive (TP) and false positive (FP) genes, positive predictive value (PPV), the actual false discovery rate (FDR), sensitivity and specificity for Med-pgQ2 and UQ-pgQ2 methods are computed with a constant multiplication value (50, 100, 200, 500, 1000 and 1 million) using MAQC2 data. In addition, we also reported the results from DESeq and TMM methods.
(DOCX)

S2 Table Evaluation of the small positive values added in read counts for Med-pgQ2 and UQ-pgQ2 given the nominal FDR≤0.05. The number of true positive (TP) and false positive (FP) genes, positive predictive value (PPV), the actual false discovery rate (FDR), sensitivity and specificity for Med-pgQ2 and UQ-pgQ2 methods are computed from the MAQC2 data. The results are reported for the choice of the small positive values added to the read counts (0.05, 0.1, 0.15, 0.20, 0.30, 0.40 and 0.50).
(DOCX)

S1 Appendix Description of six existing normalization methods, statistical modeling and the exact test. (DOCX)

S1 File The source codes in R script for the different normalization methods. (R)

S1 Datasets MAQC2 raw read counts. Raw read count maqc2 data with 2 replicates in 2 conditions extracted from mapping files using Python command line This data contain 36451 genes and the genes with zero counts in both conditions are filtered out.
(ZIP)

S2 Datasets DEGs analysis of Med-pgQ2-normalized MAQC2 data using *edgeR* package. Med-pgQ2-normalized MAQC2 data using our R codes are included.
(ZIP)

S3 Datasets DEGs analysis of UQ-pgQ2-normalized MAQC2 data using *edgeR* package. UQ-pgQ2-normalized MAQC2 data using our R codes are included.
(ZIP)

S4 Datasets DEGs analysis of DESeq-normalized MAQC2 data using *DESeq2* package. DESeq-normalized MAQC2 data using *DESeq* package are included.
(ZIP)

S5 Datasets DEGs analysis of TMM-normalized MAQC2 data using *edgeR* package. TMM-normalized MAQC2 data using *edgeR* package are included.
(ZIP)
