# Supplementary figures and images for: A comparison of per sample global scaling and per gene normalization methods for differential expression analysis of RNA-seq data
Source: PLoS One. 2017 May 1;12(5):e0176185. doi: 10.1371/journal.pone.0176185 (PMC5411036; doi:10.1371/journal.pone.0176185)

A

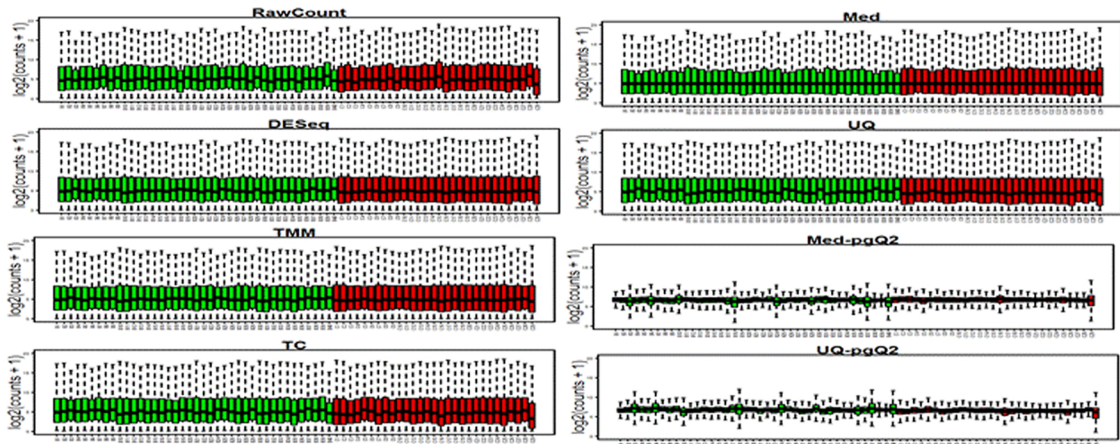

B

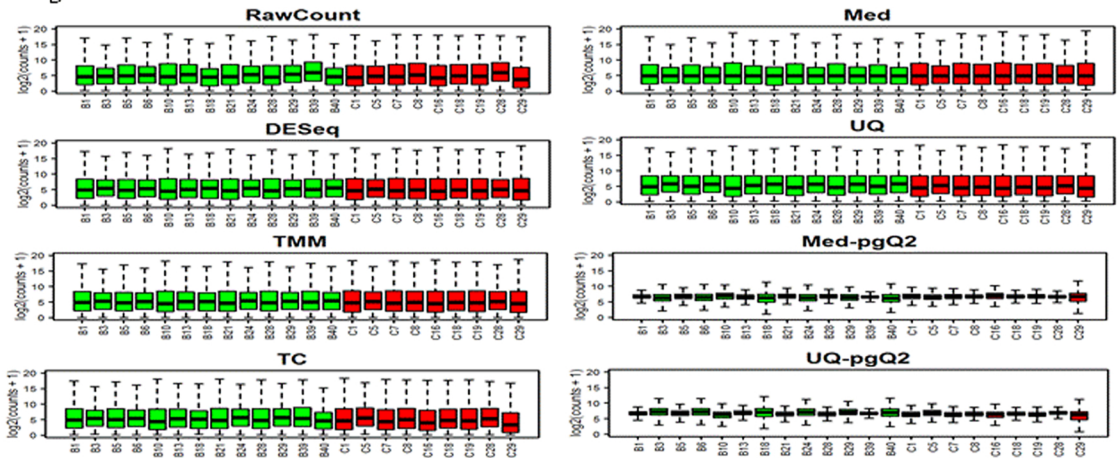

Supplement: S1 Fig — (PDF) [file pone.0176185.s001.pdf]

A

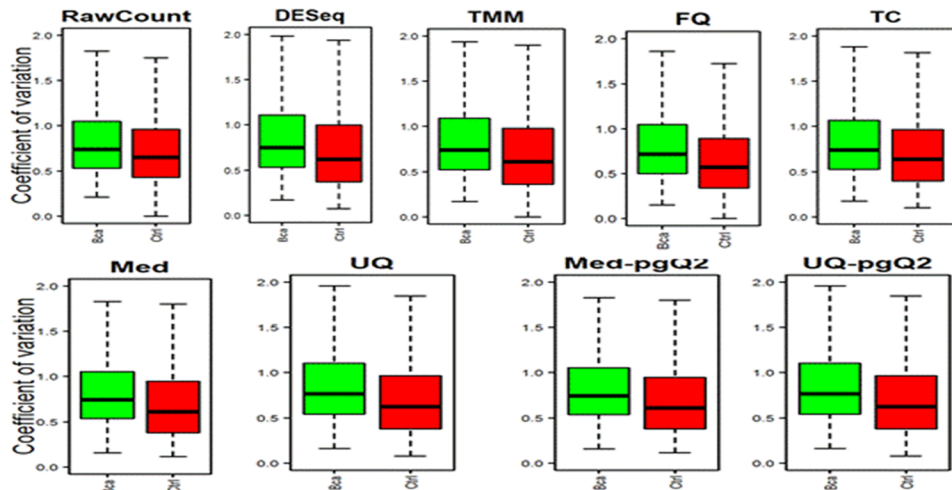

B

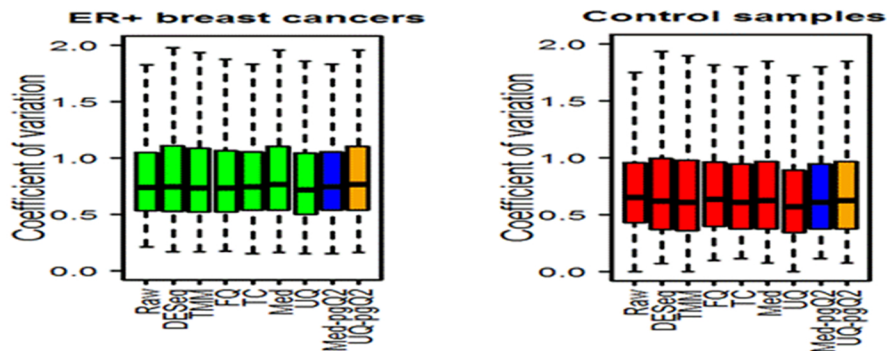

Supplement: S2 Fig — (PDF) [file pone.0176185.s002.pdf]

RawCount

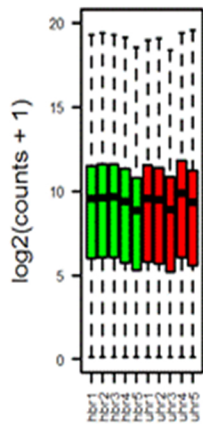

DESeq

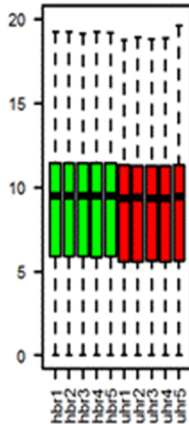

TMM

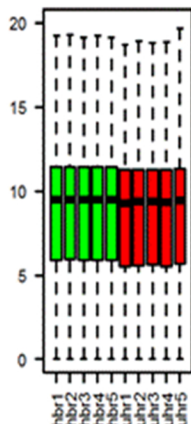

TC

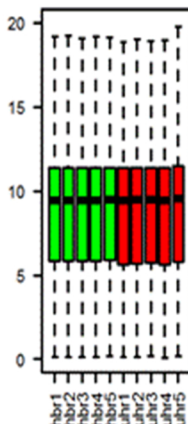

Med

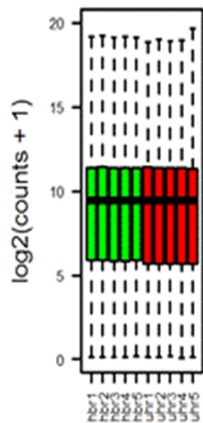

UQ

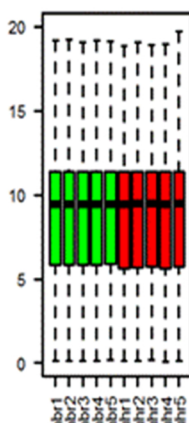

Med-pgQ2

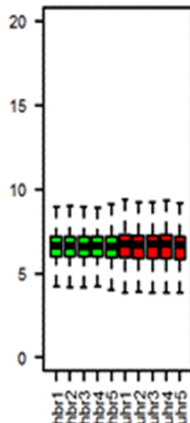

UQ-pgQ2

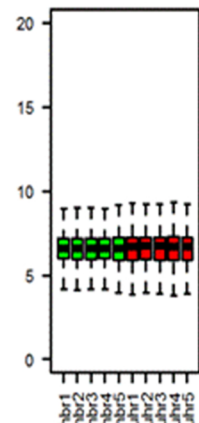

Supplement: S3 Fig — (PDF) [file pone.0176185.s003.pdf]

**UQ**

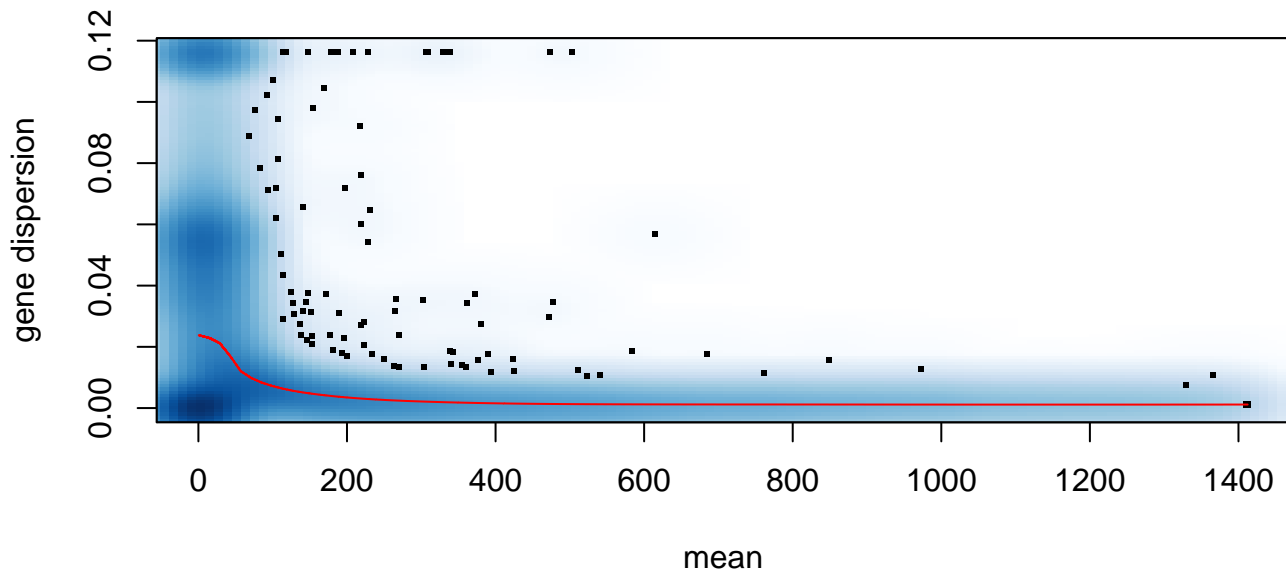

**UQ.pgQ2**

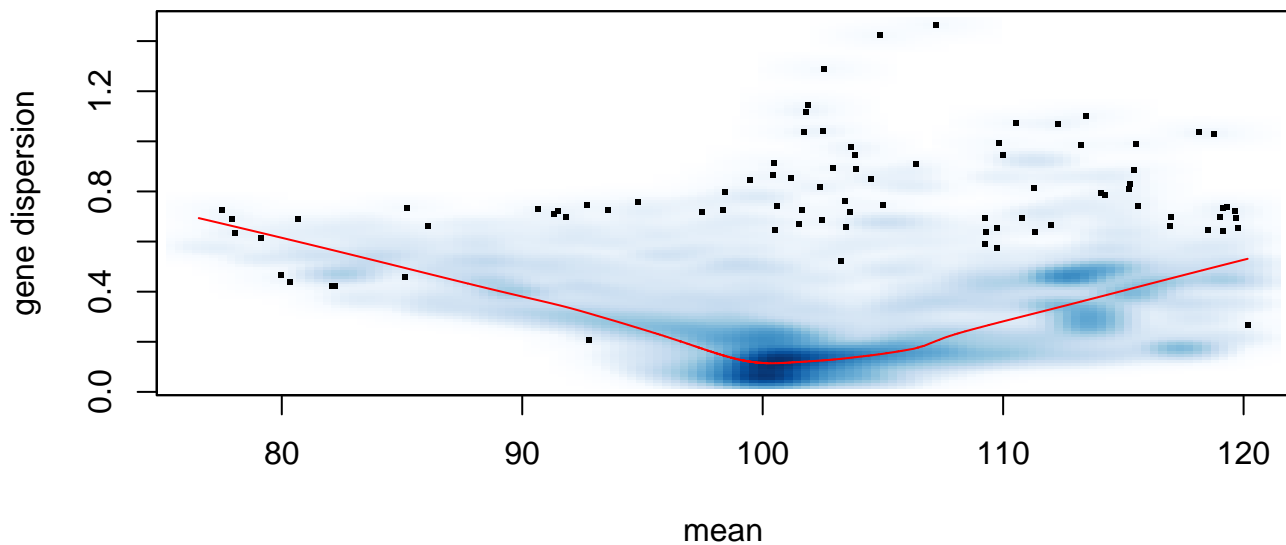

Supplement: S4 Fig — Gene-wise dispersion was estimated after UQ and UQ-pgQ2 normalization with the aid of edgeR. The top graph displays mean versus gene dispersion for genes with a quantile cutoff value of mean read count after UQ normalization of ≤ 90%, while the bottom graph displays mean versus gene dispersion for genes with a quantile cutoff value of mean read count after UQ.pgQ2 normalization of ≤ 90%. (PDF) [file pone.0176185.s004.pdf]

### Mean Count $\geq 100$

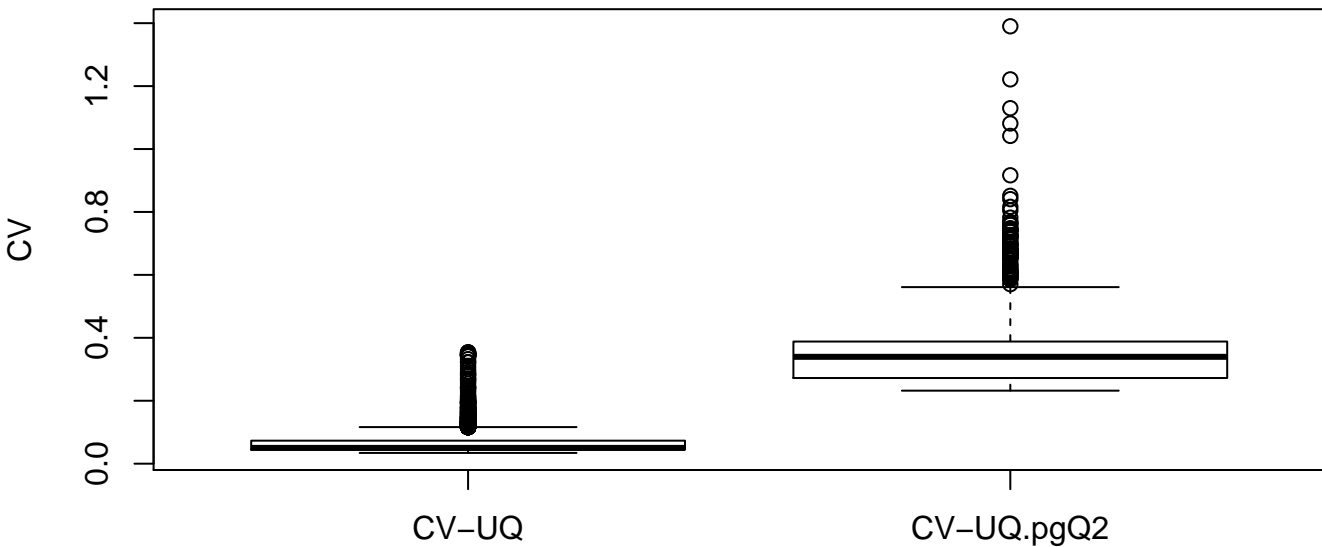

### Mean Count $< 100$

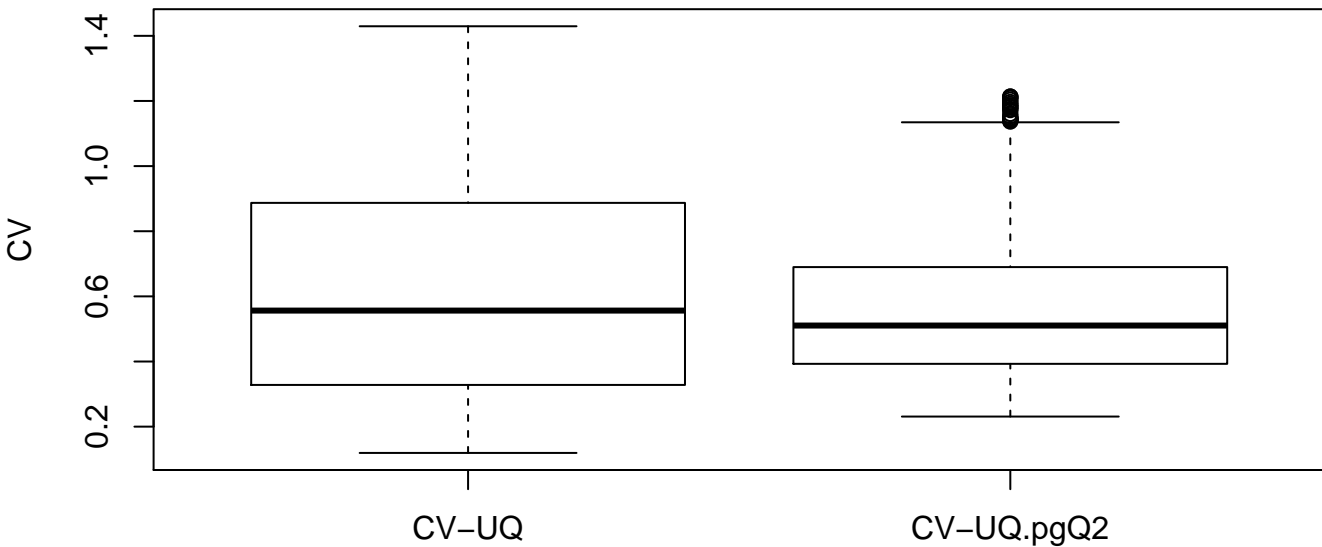

Supplement: S5 Fig — The calculated coefficient of variation (CV) after UQ normalization and per-gene (UQ.pgQ2) normalization, based on the estimated dispersion parameter from edgeR and assuming a negative binomial distribution. The top graph displays the CV for genes with mean read count after UQ normalization of ≥ 100, while the bottom graph displays the CV for genes with mean read count <100. (PDF) [file pone.0176185.s005.pdf]
