# Supplementary material for: A comparison of per sample global scaling and per gene normalization methods for differential expression analysis of RNA-seq data
Source: PLoS One. 2017 May 1;12(5):e0176185. doi: 10.1371/journal.pone.0176185 (PMC5411036; doi:10.1371/journal.pone.0176185)
Supplement: S1 Table — The number of true positive (TP) and false positive (FP) genes, positive predictive value (PPV), the actual false discovery rate (FDR), sensitivity and specificity for Med-pgQ2 and UQ-pgQ2 methods are computed with a constant multiplication value (50, 100, 200, 500, 1000 and 1 million) using MAQC2 data. In addition, we also reported the results from DESeq and TMM methods. (DOCX) [file pone.0176185.s006.docx]

**Table S1: Evaluation of constant multiplication values for Med-pgQ2 and UQ-pgQ2 given the nominal FDR**$\boldsymbol{\leq}$**0.05.** The number of true positive (TP) and false positive (FP) genes, positive predictive value (PPV), the actual false discovery rate (FDR), sensitivity and specificity for Med-pgQ2 and UQ-pgQ2 methods are computed using the MAQC2 data. These results of evaluation of the constant multiplication values (f=50, 100, 200, 500, 1000 and 1 million) are reported.

|  | Multiplication factor | # of TP genes | # of FP genes | PPV | Actual FDR | Sensitivity | Specificity |
| --- | --- | --- | --- | --- | --- | --- | --- |
| Med-pgQ2 | 50 | 365 | 23 | 0.941 | 0.059 | 0.936 | 0.848 |
|  | 100 | 362 | 22 | 0.943 | 0.057 | 0.928 | 0.850 |
|  | 200 | 362 | 21 | 0.945 | 0.055 | 0.928 | 0.861 |
|  | 500 | 361 | 20 | 0.948 | 0.053 | 0.926 | 0.868 |
|  | 1000 | 361 | 20 | 0.948 | 0.053 | 0.926 | 0.868 |
|  | ${10}^{6}$ | 360 | 20 | 0.947 | 0.053 | 0.923 | 0.868 |
| UQ-pgQ2 | 50 | 364 | 24 | 0.938 | 0.062 | 0.933 | 0.841 |
|  | 100 | 364 | 21 | 0.946 | 0.055 | 0.933 | 0.861 |
|  | 200 | 363 | 21 | 0.945 | 0.055 | 0.931 | 0.861 |
|  | 500 | 362 | 21 | 0.945 | 0.055 | 0.928 | 0.861 |
|  | 1000 | 362 | 21 | 0.945 | 0.055 | 0.928 | 0.861 |
|  | ${10}^{6}$ | 361 | 20 | 0.948 | 0.053 | 0.928 | 0.868 |
| DESeq | - | 363 | 59 | 0.860 | 0.140 | 0.931 | 0.609 |
| TMM-edgeR | - | 377 | 97 | 0.796 | 0.205 | 0.964 | 0.358 |
