# Supplementary material for: A comparison of per sample global scaling and per gene normalization methods for differential expression analysis of RNA-seq data
Source: PLoS One. 2017 May 1;12(5):e0176185. doi: 10.1371/journal.pone.0176185 (PMC5411036; doi:10.1371/journal.pone.0176185)
Supplement: S2 Table — The number of true positive (TP) and false positive (FP) genes, positive predictive value (PPV), the actual false discovery rate (FDR), sensitivity and specificity for Med-pgQ2 and UQ-pgQ2 methods are computed from the MAQC2 data. The results are reported for the choice of the small positive values added to the read counts (0.05, 0.1, 0.15, 0.20, 0.30, 0.40 and 0.50). (DOCX) [file pone.0176185.s007.docx]

**Table S2: Evaluation of the small positive values added in read counts for Med-pgQ2 and UQ-pgQ2 given the nominal FDR**$\boldsymbol{\leq}$**0.05.** The number of true positive (TP) and false positive (FP) genes, positive predictive value (PPV), the actual false discovery rate (FDR), sensitivity and specificity for Med-pgQ2 and UQ-pgQ2 methods are computed from the MAQC2 data. The results for the choice of the small positive values added the read counts (0.05, 0.10, 0.15, 0.20, 0.30, 0.40 and 0.50).

|  | Small Positive # added | # of TP genes | # of FP genes | PPV | Actual FDR | Sensitivity | Specificity |
| --- | --- | --- | --- | --- | --- | --- | --- |
| Med-pgQ2 | 0.05 | 347 | 17 | 0.953 | 0.047 | 0.890 | 0.887 |
|  | 0.10 | 362 | 22 | 0.943 | 0.057 | 0.928 | 0.850 |
|  | 0.15 | 366 | 26 | 0.934 | 0.066 | 0.938 | 0.828 |
|  | 0.20 | 369 | 30 | 0.925 | 0.075 | 0.946 | 0.801 |
|  | 0.30 | 374 | 39 | 0.906 | 0.094 | 0.946 | 0.795 |
|  | 0.40 | 377 | 45 | 0.893 | 0.101 | 0.962 | 0.722 |
|  | 0.50 | 377 | 48 | 0.887 | 0.112 | 0.967 | 0.682 |
| UQ-pgQ2 | 0.05 | 344 | 18 | 0.950 | 0.050 | 0.882 | 0.881 |
|  | 0.10 | 364 | 21 | 0.946 | 0.055 | 0.933 | 0.861 |
|  | 0.15 | 367 | 31 | 0.922 | 0.078 | 0.941 | 0.795 |
|  | 0.20 | 372 | 35 | 0.914 | 0.086 | 0.954 | 0.768 |
|  | 0.30 | 375 | 42 | 0.899 | 0.107 | 0.962 | 0.721 |
|  | 0.40 | 376 | 50 | 0.882 | 0.117 | 0.964 | 0.669 |
|  | 0.50 | 376 | 53 | 0.877 | 0.123 | 0.964 | 0.649 |
| DESeq | - | 363 | 59 | 0.860 | 0.140 | 0.931 | 0.609 |
| TMM-edgeR | - | 377 | 97 | 0.796 | 0.205 | 0.964 | 0.358 |
