# Supplementary material for: A comparison of per sample global scaling and per gene normalization methods for differential expression analysis of RNA-seq data
Source: PLoS One. 2017 May 1;12(5):e0176185. doi: 10.1371/journal.pone.0176185 (PMC5411036; doi:10.1371/journal.pone.0176185)
Supplement: S1 Appendix — (DOCX) [file pone.0176185.s008.docx]

**Appendix for “A Comparison of per Sample Global Scaling and per Gene Normalization for Differential Expression analysis of RNA-seq Data”**

Authors :Xiaohong Li^1,2^, Guy N. Brock^1,3^, Eric C. Rouchka^4^, Nigel G.F. Cooper^2^, Dongfeng Wu^1^, Timothy E. O’Toole^5^, Ryan S. Gill^6^, Abdallah M. Eteleeb^7^, Liz O’Brien^8^, Shesh N. Rai^1*^

^1^ Department of Bioinformatics and Biostatistics, University of Louisville, Louisville, KY, USA

^2^ Department of Anatomical Sciences and Neurobiology, University of Louisville, Louisville, KY, USA

^3^ Department of Biomedical Informatics, the Ohio State University, Columbus, OH, USA

^4^ Department of Computer Engineering Computer Science, University of Louisville, Louisville, KY, USA

^5^ Department of Cardiology, University of Louisville, Louisville, KY, USA

^6^ Department of Mathematics, University of Louisville, Louisville, KY, USA

^7^ Department of Internal Medicine, Oncology Division, Washington University, St. Louise, MO, USA

^8^ Department of Epidemiology, University of Louisville, Louisville, KY, USA

**1. Methods**

**1.1 Normalization methods**

In the following, the statistical notations for characterizing different normalization methodologies are defined. For simplicity, we only consider the gene $g (g=1, \ldots, G)$ in sample $j (j=1,\ldots, n)$ where *G* is the total genes and *n* is the total number of samples. Let $Y_{gj}$ be the number of observed reads mapped to a gene $g$ for sample *j,* $N_{j}$ be the total number of mapped reads for all genes in sample *j,* *N* be the total number of mapped reads across all samples, $\bar{N}$ be the mean of the reads across all samples, $u_{gj}$be the true and unknown expression level and $L_{g}$ be the length of the gene $g$.

The above $N_{j}$, *N* and $\bar{N}$can be expressed as:

$N_{j}=\sum_{g=1}^{G} Y_{gj}$, $N=\sum_{j=1}^{n} N_{j}$, and $\bar{N}=\frac{\left( \sum_{j=1}^{n} N_{j} \right)}{n}$ .

1. **Reads Per Kilobase of a transcript per Million mapped reads (RPKM) [**[**1**](#_ENREF_1)**]**

Since a long gene will have more reads mapping to it compared to a short gene of similar expression, the length of the gene was considered in the RPKM normalization. Let $Y_{gj}^{RPKM}$ be the RPKM-normalized reads of gene $g$ for sample *j*. The observed $Y_{gj}$ is scaled by both the total number of mapped reads $\left( N_{j} \right)$ per million reads and the length of the transcript $\left( L_{g} \right)$ per kilobase. Then $Y_{gj}^{RPKM}$ can be expressed as:

$Y_{gj}^{RPKM}$= $\frac{Y_{gj}}{N_{j} \times L_{g}}\times{10}^{9}$ = $\frac{Y_{gj}}{\frac{N_{j}}{{10}^{6}}\times\frac{L_{g}}{{10}^{3}}}=\frac{Reads per transcript}{\frac{Total reads}{{10}^{6}}\times\frac{transcript length}{{10}^{3}}}$. (1)

The RPKM normalization is a scaled normalization due to the different number of total reads as well as the different lengths of the gene $g$ in sample *j*.

1. **Fragments Per Kilobase of transcript per Million mapped fragments (FPKM)**

FPKM normalization is used for paired-end RNA-seq data and the relative expression of a transcript or gene is proportional to the number of cDNA fragments that originate from it. Let $Y_{gj}^{FP}$ be the FPKM-normalized reads of gene $g$ for sample *j*. The observed $Y_{gj}$ is scaled by both the total number of mapped fragments $\left( N_{j} \right)$ per million fragments and the length of the fragment of the gene or transcript $\left( L_{f} \right)$ per kilobase. Then $Y_{gj}^{FPKM}$ can be expressed as:

$Y_{gj}^{FPKM}$= $\frac{Y_{gj}}{N_{j}\times L_{f}}\times{10}^{9}$ = $\frac{Y_{gj}}{\frac{N_{j}}{{10}^{6}}\times\frac{L_{f}}{{10}^{3}}}=\frac{Fragments per transcript}{\frac{Total \# \mathrm{of}fragments}{{10}^{6}}\times\frac{fragment length}{{10}^{3}}}$. (2)

The FPKM normalization is a scaled normalization due to the different number of total fragments as well as the different lengths of the gene $g$ in sample *j*. The difference between RPKM and FPKM is that each mapped paired-end cDNA fragments will be counted as one in FPKM, but it will be counted as two reads in RPKM.

1. **Total count (TC) per sample**

Let $Y_{gj}^{TC}$ be the TC-normalized reads of gene $g$ in sample $j$. The observed $Y_{gj}$ is scaled by the total number of mapped reads $\left( N_{j} \right)$ per average of total reads across all the samples of the dataset$\left( \bar{N} \right)$. Then $Y_{gj}^{TC}$ can be expressed as:

$Y_{gj}^{TC}=\frac{Y_{gj}}{N_{j}}\times\bar{N}=\frac{Y_{gj}}{N_{j}/\bar{N}}$. (3)

A study evaluating statistical methods for normalization in RNA-seq experiments [[2](#_ENREF_2)] demonstrated that upper-quartile normalization reduced bias in the estimation of DEGs relative to qRT-PCR without noticeably increasing the level of variability as compared to total-count (TC) normalization.

1. **Trimmed Mean of M-value (TMM) [**[**3**](#_ENREF_3)**]**

Since we do not know the expression levels and true length of each transcript in RNA-seq data, RNA expression cannot be directly estimated from the raw read count. However, a relative gene expression level of two samples, i.e., a global fold change can be estimated. TMM is implemented in *edgeR* that assumes that a majority of the genes between samples are not differentially expressed. Let $Y_{gj}^{TMM}$ be the TMM-normalized reads of gene $g$ in sample *j* and $S_{j}$ be the library size which is the unknown total RNA output of sample *j*.

In RNA-seq, a gene-wise log-fold-change of sample *j* relative to the reference sample *r* can be expressed as:

$M_{gj}={log}_{2}\left( \frac{Y_{gj}/N_{j}}{Y_{gr}/N_{r}} \right)$, (4)

and absolute expression levels can be expressed as:

$A_{gj}=\frac{1}{2}{log}_{2}^{{(Y}_{gj}/N_{j}\times Y_{gr}/N_{r})}$ for $Y_{gj}\neq0$. (5)

A trimmed mean is the average after removing the upper and lower $x\%$ of the data. By default, the TMM procedure is doubly trimmed, where $M_{gj}$ values are trimmed by 30% and $A_{gj}$ values are trimmed by 5%. After trimming, a weighted mean of$M_{gj}$ is calculated and the normalization factor $f_{j}^{TMM}$ for sample *j* using reference sample $r$ is expressed as:

$f_{j}^{TMM}=\frac{\sum_{g\in G^{*}} w_{gj}{\times M}_{gj}}{\sum_{g\in G^{*}} w_{gj}}$, where $M_{gj}={log}_{2}\left( \frac{Y_{gj}/N_{j}}{Y_{gr}/N_{r}} \right),$ (6)

and $w_{gj}=\frac{N_{j}-Y_{gj}}{N_{j}{\times Y}_{gj}}+\frac{N_{r}-Y_{gr}}{N_{r}\times Y_{gr}}$ , where $Y_{gj}>0$and $Y_{gr}>0$.

$G^{*}$represents the set of genes with valid $M_{gj}$ and $A_{gj}$ values, which is trimmed in advance of the calculation of $M_{gj}$ and $A_{gj}$ since log-fold-change cannot be calculated with zero transcript or gene reads in any selected samples $\left( Y_{gj} \right.=0$or $Y_{gr}=0)$. Therefore, $G^{*}$ is not trimmed by the percentage above. Normalization factors across several samples can be calculated by selecting one sample as a reference and calculating the TMM factor for each non-reference sample. For example, for a two-sample comparison, only one relative scaling factor $\left( f_{j}^{TMM} \right)$ is required. It can be used to adjust both observed library sizes such as:

$S_{j}^{TMM}=S_{j}\times\sqrt{f_{j}^{TMM}}$ and $S_{r}^{TMM}=S_{r}/\sqrt{f_{j}^{TMM}}$, (7)

where $S_{j}=\sum_{g=1}^{G} u_{gj}\times L_{g}$, $S_{r}=\sum_{g=1}^{G} u_{gr}\times L_{g}$. In fact, $u_{gj}$ and $u_{gr}$ are the true and unknown expression level in sample *j* and *r*, respectively. They are defined as:

$u_{gj}=\frac{E(Y_{gj})S_{j}}{L_{g}N_{j}}$ and $u_{gr}=\frac{E(Y_{gr})S_{r}}{L_{g}N_{r}}$, where $Y_{gj}$ and $Y_{gr}$ are the observed count for gene *g* in sample *j* and reference sample *r*, respectively.

The normalization and scaling factors provided by the *calcNormFactors*() function in the *edgeR* Bioconductor package are rescaled by the mean of the normalized library sizes as [[4](#_ENREF_4)]:

$\bar{S}^{TMM}=\frac{\sum_{j=1}^{n} S_{j}^{TMM}}{n}$. (8)

$Y_{gj}^{TMM}$ is obtained using a re-scaled normalization factor to scale the raw read counts [[4](#_ENREF_4)]:

$Y_{gj}^{TMM}=\frac{Y_{gj}}{f_{j}^{TMM}/\bar{S}^{TMM}} \{Dillies, 2013 \#149\}$. (9)

One notable difference with TMM normalization for RNA-seq is that the data themselves do not need to be modified and the estimated normalization factors are directly used in the statistical model to test for differentially expressed genes, while preserving the sampling properties of the data.

1. **DESeq normalization [**[**5**](#_ENREF_5)**]**

Although the DESeq normalization method was originally developed by the DESeq package, we use the DESeq normalization (median-of-ratios) and a Wald test statistic for detecting DEGs in DESeq2 package[[6](#_ENREF_6)]. The DESeq normalization is summarized as follow.

Like TMM, *DESeq* normalization is based on the assumption that most of the genes are not DE. Let $Y_{gj}^{DESeq}$ be the DESeq-normalized reads of gene $g (g=1, \ldots, G)$ in sample *j*. A DESeq size factor $f_{j}^{DESeq}$given a sample $j$ is calculated as the median of the ratios for each gene. The $f_{j}^{DESeq}$ can be expressed as:

$f_{j}^{DESeq}=\underset{g}{\mathrm{median}} \frac{Y_{gj}}{\left( \prod_{j'=1}^{n} Y_{gj'} \right)^{1/n}}$ , (10)

where the denominator of this expression can be interpreted as a pseudo-reference sample obtained by taking the geometric mean across samples. Then $Y_{gj}^{DESeq}$ is obtained by scaling the raw reads $Y_{gj}$ by $f_{j}^{DESeq}$

$Y_{gj}^{DESeq}=\frac{Y_{gj}}{f_{j}^{DESeq}}$. (11)

1. **Full quantile normalization (FQ) [**[**7**](#_ENREF_7)**,**[**8**](#_ENREF_8)**]**

The full quantile normalization was originally used for the normalization of Affymetix GeneChip and one-color cDNA microarray [[8](#_ENREF_8)]. The goal of quantile normalization is to make the distribution of probe intensities the same for arrays $j=1,\ldots, n$, so that an *n*-dimensional quantile-quantile plot follows the *n*-dimensional identity line. The quantile normalization method of RNA-seq data consists of matching the distributions of gene or transcript reads across samples.

Let $Y_{gj}^{FQ}$ be the quantile-normalized reads of gene $g$ in sample *j*. Let $q_{g}=\left( q_{1g},\ldots, q_{ng} \right)$ be the vector of $g$th quantile for $n$ arrays and $d=\left( \frac{1}{\sqrt{n}}, \ldots, \frac{1}{\sqrt{n}} \right)$ be the unit diagonal. The projection of *q* onto *d* is obtained by $\underset{d}{\mathrm{proj}} q_{g}=\left( \frac{1}{n}\sum_{j=1}^{n} q_{gj},\ldots,\frac{1}{n}\sum_{j=1}^{n} q_{gj} \right)$. This implies that each sample can be given the same distribution by taking the mean quantile and substituting it as the value of the data item in the original dataset. The algorithm for normalizing a set of data vectors by giving them the same distribution is described as follows. 1) Let $Y_{gj}$ be the raw reads of genes. A matrix of *Y* is formed with *G* x *n* dimension given *n* samples and *G* genes; 2) Sort each column of *Y* to give $Y_{sort}$; 3) Take the mean across rows of $Y_{sort}$ to obtain the mean reads per row and assign the mean to each row across samples to get $Y_{sort}^{'}$; 4) Getting the normalized $Y^{QT}$by rearranging each column of $Y_{sort}^{'}$ to have the same ordering as original *Y*.

One possible problem with this method is that it forces the values of the quantiles to be equal. Therefore, it risks removing some of the signal in the tails. However, in practice, since gene expression measures are typically computed using the value of multiple transcripts, this may be not a problem for the gene in RNA-seq data.

**1.2 Statistical model and the exact test**

**The negative binomial distribution:**  In general, a negative binomial random variable *Y* is defined as $Y\sim NB\left( p,r \right)$, which counts the number of failures before the $r^{th}$ success in a series of independent and identical Bernoulli trials with probability of success *p*. However, an alternative parametrization is widely used to model RNA-seq data denoted $Y\sim NB\left( u,ф \right)$, where the random variable *Y* has mean *u* and dispersion *ф*. Its probability mass function (pmf), expected value and variance of *Y* are defined as:

$f_{Y}\left( y|u,ф \right)=P \left( Y=y \right)=\left( \begin{matrix} y+ф^{-1}-1 \\ y \end{matrix} \right)\left( \frac{1}{uф+1} \right)^{ф^{-1}}\left( 1-\frac{1}{uф+1} \right)^{y},$ (12)

$E\left( Y \right)=u,\mathrm{and} \mathrm{Var}\left( Y \right)=u+u^{2}ф$.

The relationship of the parameters can be expressed as:

$u=r\frac{1-p}{p} \mathrm{and} ф=\frac{1}{r}$. (13)

Moreover, a negative binomial distribution can be derived from a Poisson-gamma mixture and hierarchy as:

$$Y|\lambda\sim Poisson\left( \lambda\right) and \lambda\sim gamma\left( ф^{-1}, uф \right).$$

Then the marginal distribution of *Y* is a negative binomial and its pmf is expressed as the equation (12) [[9](#_ENREF_9)].

**Conditional dispersion estimation [**[**10**](#_ENREF_10)**]:** In this study, *edgeR* was used to evaluate the normalization methods. Since all libraries have the same library size after normalization (size factor equal to one), a CML (conditional maximum likelihood) is used in *edgeR* to estimate the dispersion parameter ($ф_{g}$) for a single gene *g* and sample *j* in *n* samples which is denoted as $Z=\sum_{j}^{n} Y_{gj}\sim NB\left( nu, \frac{ф_{g}}{n} \right)$. It is expressed as:

$l_{y|Z=z}\left( ф_{g} \right)=\left[ \sum_{j}^{n} \log\Gamma\left( y_{gj}+ф_{g}^{-1} \right) \right]+\log\Gamma\left( nф_{g}^{-1} \right)-\log\Gamma\left( z+nф_{g}^{-1} \right)-n\log\Gamma\left( ф_{g}^{-1} \right).$ (14)

**Exact test for a two-condition comparison in RNA-seq:** The exact test for DEGs between two conditions is the best choice for RNA-seq [[10](#_ENREF_10)]. Both *edgeR* [[11](#_ENREF_11)] and *DESeq* implement similar exact test [[5](#_ENREF_5),[9](#_ENREF_9)]. Briefly, let $Y_{gij}$ be the normalized counts of gene *g* in condition *i* = A and B, and replicate $j=1,\ldots,n_{i}$*.* Then the assumptions concerning the distributions of $Y_{gij} \mathrm{and} \sum_{j}^{n_{i}} Y_{gij}$ are expressed as:

$Y_{gij}\sim NB(u_{gi}\cdot s_{ij}$, $\frac{ф_{g}}{s_{ij}}$) ≡ $Y_{gij}\sim NB(u_{gi}$,$ф_{g}$), where size factor $s_{ij}=1$ for normalized counts,

$\sum_{j}^{n_{i}} Y_{gij}\sim NB(u_{gi}\cdot\sum_{j=1}^{n_{i}} s_{ij}$, $\frac{ф_{g}}{\sum_{j=1}^{n_{i}} s_{ij}}$) ≡ $\sum_{j}^{n_{i}} Y_{gij}\sim NB\left( n_{i}\cdot u_{gi}, \frac{ф_{g}}{n_{i}} \right), E\left( \sum_{j}^{n_{i}} Y_{gij} \right)=n_{i}\cdot u_{gi}$, and Var$\left( \sum_{j}^{n} Y_{gij} \right)=n_{i}\cdot u_{gi}+n_{i}\cdot u_{gi}^{2}\cdotф_{g}$. (15)

In order to identify DEGs between conditions A and B, we would like to test the null hypothesis $H_{0}: u_{gA}=u_{gB}$, where the test statistics are the total normalized counts in each condition, $Y_{gA}=\sum_{j}^{n} Y_{gAj}$, $Y_{gB}=\sum_{j}^{n} Y_{gBj},$ and total counts of two conditions $Y_{gS}=Y_{gA}+Y_{gB}$. The respective distributions of $Y_{gA} \mathrm{and}Y_{gB}$ are expressed as:

$Y_{gA}\sim NB\left( n_{A}\cdot u_{gA}, \frac{ф_{g}}{n_{A}} \right), \mathrm{and} Y_{gB}\sim NB\left( n_{B}\cdot u_{gB}, \frac{ф_{g}}{n_{B}} \right)$. (16)

Since $Y_{gA} \mathrm{and} Y_{gB}$ are independent, the joint probability of $P(Y_{gA}=y_{gA},Y_{gB}=y_{gB} )$ under $H_{0}$ is $P(Y_{gA}=y_{gA})\times P(Y_{gB}=y_{gB})$, In an exact test the p-value [[5](#_ENREF_5)] is calculated by summation of the probability of a pair of *P(a, b)* that is less than or equal to the observed *P(*$y_{gA},y_{gB}$*)* given that the overall summation of *P(a, b)*. The pair of variables *a* and *b* are defined as $a=0,\ldots,Y_{gS} and b=Y_{gS}-a .$ Then the p-value for gene *g* is

${p.value}_{g}=P_{1g}/P_{2g}$, (17)

where

$P_{1g}=\sum_{\begin{aligned} a+b=Y_{gS} \\ P(a,b)\leq P(y_{gA},y_{gB}) \end{aligned}} P(Y_{gA}=a)\times P(Y_{gB}=b)$,

and

$P_{2g}=\sum_{a+b=Y_{gS}} P(a, b)$. (18)

The p-value is further adjusted by a false discovery rate correction.

**2. References**

1. Mortazavi A, Williams BA, McCue K, Schaeffer L, Wold B (2008) Mapping and quantifying mammalian transcriptomes by RNA-Seq. Nat Methods 5: 621-628.

2. Bullard JH, Purdom E, Hansen KD, Dudoit S (2010) Evaluation of statistical methods for normalization and differential expression in mRNA-Seq experiments. BMC Bioinformatics 11: 94.

3. Robinson MD, Oshlack A (2010) A scaling normalization method for differential expression analysis of RNA-seq data. Genome Biol 11: R25.

4. Dillies MA, Rau A, Aubert J, Hennequet-Antier C, Jeanmougin M, et al. (2013) A comprehensive evaluation of normalization methods for Illumina high-throughput RNA sequencing data analysis. Brief Bioinform 14: 671-683.

5. Anders S, Huber W (2010) Differential expression analysis for sequence count data. Genome Biol 11: R106.

6. Love MI, Huber W, Anders S (2014) Moderated estimation of fold change and dispersion for RNA-seq data with DESeq2. Genome Biol 15: 550.

7. Bolstad BM, Irizarry RA, Astrand M, Speed TP (2003) A comparison of normalization methods for high density oligonucleotide array data based on variance and bias. Bioinformatics 19: 185-193.

8. Irizarry RA, Hobbs B, Collin F, Beazer-Barclay YD, Antonellis KJ, et al. (2003) Exploration, normalization, and summaries of high density oligonucleotide array probe level data. Biostatistics 4: 249-264.

9. Yu D, Huber W, Vitek O (2013) Shrinkage estimation of dispersion in Negative Binomial models for RNA-seq experiments with small sample size. Bioinformatics 29: 1275-1282.

10. Robinson MD, Smyth GK (2008) Small-sample estimation of negative binomial dispersion, with applications to SAGE data. Biostatistics 9: 321-332.

11. Robinson MD, McCarthy DJ, Smyth GK (2010) edgeR: a Bioconductor package for differential expression analysis of digital gene expression data. Bioinformatics 26: 139-140.
